# Validation of a lithium-ion commercial battery pack model using experimental data for stationary energy management application

**DOI:** 10.12688/openreseurope.14301.1

**Published:** 2022-02-03

**Authors:** Ana Foles, Luís Fialho, Pedro Horta, Manuel Collares-Pereira

**Affiliations:** 1 Renewable Energies Chair, University of Évora, Pólo da Mitra da Universidade de Évora, Edifício Ário Lobo de Azevedo, 7000-083 Nossa Senhora da Tourega, Portugal; 2 Institute of Earth Sciences, University of Évora, Rua Romão Ramalho, Évora, 7002-554, Portugal

**Keywords:** Electrical energy storage, lithium-ion battery, characterization tests, battery model

## Abstract

**Background:** A cost-effective solution for the design of distributed energy storage systems implies the development of battery performance models yielding a suitable representation of its dynamic behaviour under realistic operation conditions.

**Methods:** In this work, a lithium-ion battery (LIB) is tested to be further modelled and integrated into an existing energy management control system. This specific LIB (5.0 kW /9.8 kWh) is integrated with a commercial inverter and  solar photovoltaic (PV) system (3.3 kWp) as part of a microgrid that is also encompassing other storage technologies at the University of Évora, Pole of INIESC – National Research Infrastructure for Solar Energy Concentration. The battery and the inverter are fully characterized through the implementation of a testing protocol aiming at better describing the battery performance. Then, a battery model is built upon both the existent LIB description and experimental fitting regression, for real-time predictive optimization control development. Considering the pre-determined efficiency of the inverter, the model allows to obtain the voltage curve, the series resistance (i.e., to describe instantaneous voltage drop/rise and transients), and the state of charge (SOC) and/or energy capacity, based on the current input. The developed model is validated through the comparison with the experimental results.

**Results:** In discharge state, the model approach presented a higher voltage RMSE (root mean square error) of 5.51 V and an MRE (maximum relative error) of 5.68 %. Regarding SOC the MRE obtained was approximately 6.82 %. In charge state, the highest RMSE voltage was 5.27 V, with an MRE of 6.74 %. Concerning SOC, the MRE obtained was approximately 6.53 %.

**Conclusions:** The developed setup allowed us to perform the necessary characterization tests under real operating conditions. Based on computational effort, simplicity of use, and the associated model error compared with the experimental data, generally, the model describes the battery behaviour.

## Nomenclature

**Table:** 

*Abbreviation*	*Definition*
BESS	Battery Energy Storage Systems
BMS	Battery Management System
DOD	Depth of Discharge
DSM	Demand Side Management
DSO	Distributor System Operator
ECM	Electric Circuit Model
EES	Electrical Energy Storage
EMS	Energy Management Strategy
ESS	Energy Storage System
EV	Electric Vehicle
LIB	Lithium-ion Battery
R&D	Research and Development
RES	Renewable Energy Sources
SOC	State of Charge (%)
TSO	Transmission System Operator
VRE	Variable Renewable Energy

## 1. Introduction

In 2019, 2.9 gigawatts (GW) of energy storage worldwide were added, approximately 30 % less than the energy storage added in 2018. The result is justified by the early maturity stage of some energy storage technologies – with presence in a few specific markets – and by being highly dependent on support by appropriate policies ^1^. In the electrical sector, the energy storage can be used in a variety of applications, namely, to meeting the demand and for reliability in the grid peak hours or as an asset on the liberalized electricity markets. It can benefit from price arbitrage depending on the fluctuation in spot prices, from capacity credit as transmission congestion relief or resource suitability. It can serve ancillary resources as voltage or frequency regulation, and spinning or non-spinning reserves ^2^. At a smaller scale, these storage technologies can also benefit the consumer, namely, for solar photovoltaic (PV) self-consumption maximization, in the electricity shift from low demand to peak times, in the stabilization of intermittent renewable energy sources (RES), in the demand-side management (DSM) or flexible power demand, and in the smart-charging of electric vehicles (EVs) ^3^. Energy storage comprises different technologies, and in this work, the electrical sector application of a lithium-ion electrochemical energy storage technology will be discussed.

Lithium-ion battery (LIB) continues to be the most deployed electrical energy storage (EES) technology, driven mainly by the downward trend in costs ^4, 5^. Characterized by a) high efficiencies, b) moderated lifetime, c) low volume and weight per kWh of storage, d) temperature sensibility, and by being associated with low maintenance compared to other battery technologies, LIB are the state-of-art technology for electric vehicles (EVs) with across-the-globe investments by large market player battery manufacturers ^5^. It is a mature technology in the mobile device market, currently being deployed in the automotive sector, and is in early market stage in stationary applications. Nowadays, the automotive market is ten times greater than the grid-scale market. The research and development (R&D) efforts made and the diminishing costs of the EV’s batteries could boost the commercial and residential market. The search for alternative battery chemistries (post-lithium) to allow for better performance (power/energy rates for example), could also offer a solution for the market, with possible declining battery costs. The ongoing scale-up allowed LIBs to present a downward trend regarding costs in the past years ^1, 4^, and they are forecasted to reach a cost potential of 70$/kWh in 2050 ^5^. The optimized cost reduction path goes along the further improvements of energy and economic indicators, such as the energy density, but also along the industrialization value chain.

This work is focused on the LIB characterization testing and modelling for real-time application on future battery control scenarios and energy management strategies. In the literature, the modelling of LIB for EV application is broad, but for stationary applications, e.g., solar photovoltaic systems, (validated with experimental results) the scientific literature found is scarce. This work aims to use a model to represent the dynamic behaviour of the battery with an adequate error, considering the interface with the power electronics, and validating it through results experimentally obtained, to allow the possibility of model integration into a more complex control system or algorithm. In the medium term, the developed model will later be integrated with models of other storage technologies and will allow optimizing the control and global operation of a complex, but flexible and intelligent grid system, that will allow it to respond to the objectives of the electrical networks of the future.

This paper is organized as follows: Section 2 delivers a bibliographic literature review on lithium-ion battery technology and the existing modelling approaches that better describes the LIB in focus in this work. Section 3 presents the methodology, namely, the experimental microgrid description, the LIB and inverter characterization testing plan, and the detailed description of the used model. Section 4 presents the results obtained, including the experimental data and the battery modelling results. Based on the methods used and the results obtained, a discussion is carried out in Section 5, followed by the conclusions of the work in Section 6.

## 2. Literature review

In this section, the lithium-ion technology is briefly reviewed, as well as the technology modelling approaches that are described throughout the bibliographic references.

### 2.1 The lithium-ion technology

The lithium-ion battery (LIB) was conceived and developed by the Japanese Asahi Kasei Corporation and released commercially in 1991 by Sony Corporation, followed by A&T Battery Co. in 1992 ^6^, with focus on low-power portable applications. The technology was well accepted given its characteristics of high-energy-density, good performance, less heat generation, small dimension, lightweight (Wh/kg) ^7^, and no memory effect, when compared with nickel-cadmium or nickel-hydride batteries. The low atomic number of lithium is the cause of the high electrode potential, which results in higher energy density. Over 90% of the worldwide production of LIBs is based in Japan, Korea and China ^8^.

The development of new high-energy-density lithium batteries has been challenging, requiring new anodes, cathodes, and nonaqueous electrolytes ^6^. Generally, a lithium-ion battery has two electrodes and an organic electrolyte, is nonaqueous, and contains dissolved lithium salts. The cathode is of lithium metal oxide and the anode is from graphitic carbon. Inside the cell, the materials are ionically and not electrically connected by an electrolyte, and it has an insulating membrane. The reaction occurs with a characteristic electrochemical potential difference (voltage). LIB cathode materials can be associated with a variety of multiple chemistries, as lithium cobalt oxide (LCO), lithium nickel manganese cobalt oxide (NMC), lithium manganese oxide (LMO), nickel cobalt aluminium oxide (NCA), and lithium iron phosphate (LFP). Recently, besides graphite, the anode can be composed of lithium titanate (LTO). NMC is the more typical chemistry for use in grid-scale ESS ^9^. Overall characteristics depend on the cell chemistry, although, generally it has higher gravimetric and volumetric energy density compared to other battery technologies, high efficiency, high power capability, long cycle, and long calendar lifetime ^10^.

LIBs allow fast and slow charging-discharging operation states, have high energy densities, and have reasonable power densities. The technology has a battery management system (BMS) which also monitors the general operating conditions (range previously defined by the manufacturer), given its sensibility to high-temperature operation and high depth of discharge (DOD), usually linked to faster degradation conditions (ageing), permanent damage, or unsafe operation. The response time of this technology is usually in the millisecond’s timescale, a fast response compared to the average of other battery technology types. It is also easily scalable in terms of power or energy. In recent years, R&D has evolved with the use of non-flammable and or flame-retardant additives (non-flammable electrolytes) ^6^. LIBs are highly sensitive to temperature. Usually, an active cooling system is integrated within the building/container of the battery (or in the EV refrigeration battery system) to reach its optimal temperature range (or move away from extreme temperature ranges). Generally, LIBs are designed to operate at temperatures about 21°C, so a heating-cooling active system can be used.

Improvements of this technology are as a result of its use in energy storage. Its market price is competitive, as it’s still associated with high production costs’.

The technology still presents challenges in its 2 ^nd^ life usage or at the end-of-life /recycling process, because its salvage value is lower than the processing cost ^11^. After high-intensity applications, as in EVs application, LIBs will generally be in good condition to be further used in high energy density applications, such as in grid storage ^12, 13^. In the present, LIB recycling is limited, having recycling figures below 3% ^14, 15^, but this will be a vital issue in its future deployment. Different LIB technologies are recycled through similar processes to recover materials like lithium, copper, cobalt, nickel, iron, aluminium, and manganese. The level of toxicity of the substances used in LIB is lower than other battery technologies, but in some countries these are still disposed of in landfills ^15^. For LIBs, lithium appears to be the only critical raw material, while other critical elements are being studied in order to reduce their need, e.g. manganese instead of critical cobalt is expected to be used for electrode coatings in the future ^16^.

***2.1.1 Standards for battery operation***

The following standards are the currently most relevant in force for the technology:

▪ UL 1642 ^17^ is a standard that expresses guidelines for manufacturers, with procedures on construction, performance, and electrical, mechanical, environmental, and marking tests.
▪ LIB’s transport guidelines are described in IEC 62281 and ST/SG/AC.10/27/ ^18^, with the United Nations recommendations.

And the general standards for secondary batteries are highlighted in the following:

▪ For household and commercial batteries, UL 2054 ^19^ is a reference for understanding the considerations made for residential sector application, regarding construction and testing for the electrical, mechanical, enclosure, fire, and environmental performance and conditions, and marking.
▪ IEC 61427-1 and IEC 61427-2 refer to the photovoltaic off-grid application and on-grid application, respectively, and IEC 62933-5-2 describes the safety requirements for grid integrated EES systems – electrochemical based systems ^18^.

Policies and the regulatory framework for batteries are still under development. For instance, the European Parliament is currently preparing the proposal for a regulation on batteries and waste batteries, including raw materials, carbon footprint and end-of-life handling, setting the sustainability requirements for EES technologies ^20^. The regulation is missing for different application scenarios of the lithium-ion battery. For the relevance of this work, key aspects on the integration, testing and operation (BMSs, safety measures, among other) of the specific technology.

### 2.2 Modelling a lithium-ion battery

LIB electric models are being emphasized in the current battery’s panorama ^21– 25^, most of them developed due to the automotive sector usage ^26– 28^. A battery model predicts the performance of the battery unit, to be used on a simulation framework, allowing the optimization of the system itself as well as its integrated control within a microgrid. Generally, it starts at a single-cell level, progressing to a unit system description, using the characteristics of nominal capacity and voltage. The efficiency losses due to racking, hardware for control and safety, and power converter elements should be considered as well. The voltage curve depends on the battery state of charge, operating current, resistance, and energy capacity. The internal resistance of the electric model increases with age, conducting to a decreasing usable voltage range. Operating the battery at higher currents is related to higher rate of voltage decrease, reducing the available bulk capacity. The thermal behaviour could be described using a heat transfer coefficient with the environment for instantaneous thermal effects on capacity and resistance, and the temperature effect is usually described as a function of ambient temperature and operating current, resulting in a resistance parameter.

A brief review of available LIB models was made, and different approaches were found. In the article ^29^, a description of LIB models and parameter identification techniques is made; the authors of 22 developed a comparison of different LIB models; in reference 28 the authors present LIB models for automotive applications, and the authors of 21 provide a review of models for generic batteries. Modelling approaches based on MATLAB/Simulink are presented in the studies ^27, 30, 31^. Testing based on pulse-charging is carried by the authors of 32, and by the authors of 33. In the case of model approaches with emphasis on chemical modelling, the authors of 34 explored parameters identification techniques for a LIB model, in 24 an extended Kalman filter is used to estimate SOC of a LIB, and the authors of 35 estimate parameters of the electric model, as the resistances and capacitors, directly from measurements.

LIB models for automotive applications in the literature outnumber those for stationary applications. The voltage equation modelling is based on the charging and discharging efficiency, and three main approaches are found in the literature: Shepherd’s battery model, the electric circuit model, and the modified Shepherd’s battery model. These three approaches are briefly presented below.

**Figure 1.**
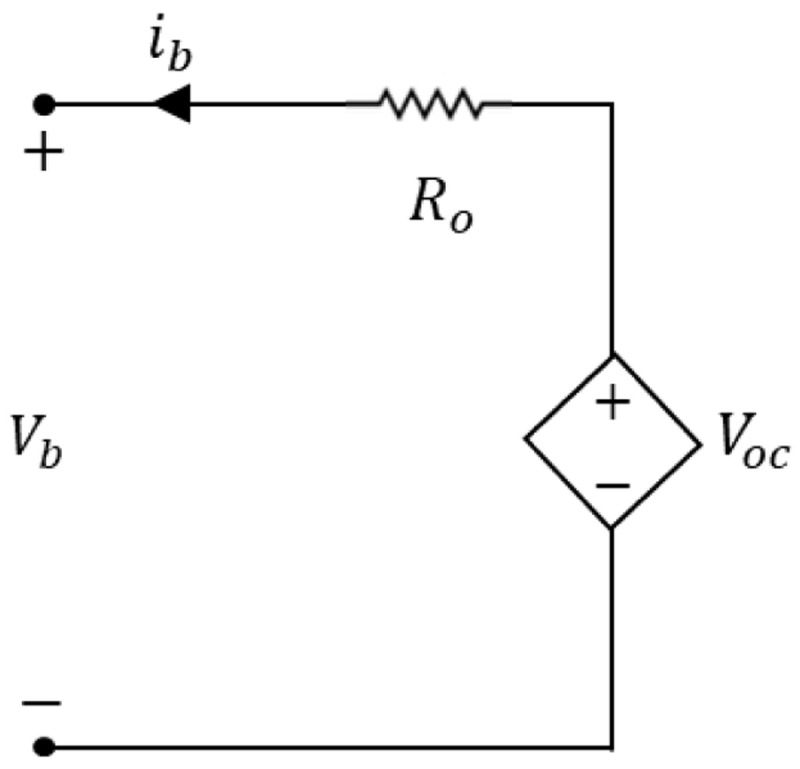
Equivalent circuit representation to the lithium-ion battery model. The current *i _b_* represents the dynamic internal current, *R _o_* represents the ohmic internal resistance, *V _b_* is the battery voltage in its terminals, and *V _oc_* is the applied input voltage.

***2.2.1. Electric circuit models (ECMs).*** Equivalent electric circuit models can be applied to describe different battery technologies ^21^. The ECMs for lithium-ion batteries found in the literature review showed satisfactory output results, following simple and fast algorithms, and able to represent the battery in permanent and transient states ^27, 31, 35^. Their suitability for stationary applications is considered a good approach. The model shown in Figure 1 has a constant voltage source in series with a resistor, and it is the simplest ECM representation.

The current *i _b_* represents the dynamic internal current, *R _o_* represents the ohmic internal resistance, *V _b_* is the battery voltage in its terminals, and *V _oc_* is the applied input voltage ^21^. The battery voltage is obtained through the simple circuit analysis of this equivalent circuit, expressed in Equation (1).


$$\;V_{battery}(t)=V_{oc}-R_{o}i_{b}(t)\;(1)$$

Among the ECMs, it is possible to find simple models, Thevenin-based models, impedance-based models, combined ECM and generic based models ^21^. The presented model includes the determination of the SOC as a function of the open-circuit voltage (V _OC_), and others could also include bias effects, and although more accurate, capacitors represent significant additional computational times.

***2.2.2. Shepherd’s battery model.*** The Shepherd’s battery model is a widely known model which describes the terminal voltage of a battery over the current inputs. It is generally described through a constant current discharge, expressed in Equation (2) ^27^,


$$\;V_{b}(t)=E_{0}-K\frac{Q}{Q-it}i(t)-R_{o}i(t)\;(2)$$

Where,

*V _b_* is the terminal voltage of the battery, in V, at instant *t*.

*E*_0_ is the battery constant voltage, in V.

   K is the polarization constant, in *V*/ *Ah*.

   Q is the battery energy capacity, described in units of Ah.

*it* is the discharge energy capacity, in Ah.

*R*_0_ is the battery internal resistance, in Ω.

*i*( *t*) is the dynamic current (A) in instant *t*.

**Figure 2.**
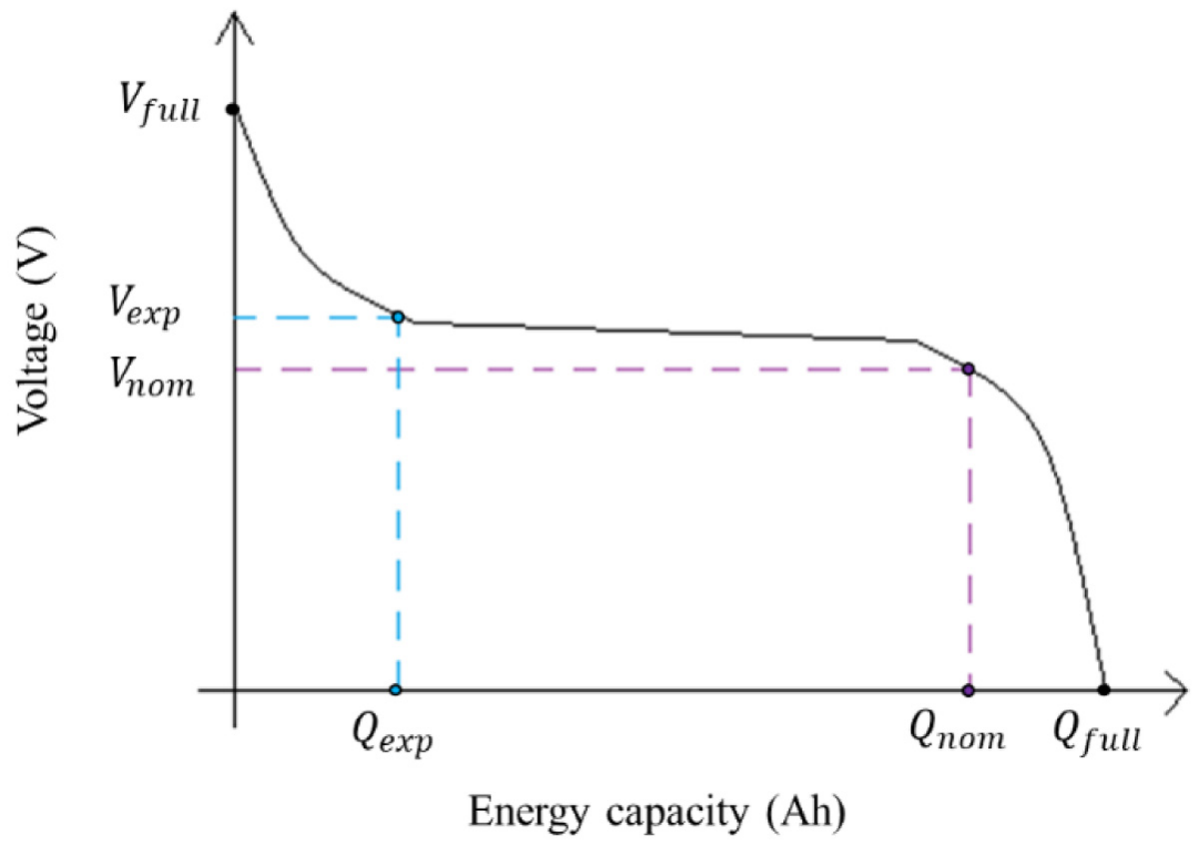
Example of an ideal discharge curve, where the three points can be extracted: the ( *V _full_*, *Q _full_*) are the voltage (V) and energy capacity (Ah) of the completely charged battery; the ( *V _exp_*, *Q _exp_*) are the voltage (V) and energy capacity (Ah) in the end of the exponential zone; and ( *V _nom_*, *Q _nom_*) are the voltage (V) and energy capacity (Ah) in the end of the nominal zone. This image has been reproduced with permission from 27.

The voltage equation parameters – *E*_0_, *K*, *R*_0_ – can be obtained through the relation established on three points of the battery discharge curve given by the manufacturer (( *V _full_*, *Q _full_*), ( *V _exp_*, *Q _exp_*) and ( *V _nom_*, *Q _nom_*)), illustrated in Figure 2, below shown.

Shepherd’s model is usually studied in the literature to describe the automotive batteries behaviour.

***2.2.3. Shepherd model modified versions.*** Shepherd’s battery model can be modified to better describe the dynamic battery behaviour through an exponential function, previously presented in the work developed by the authors of ^31^. The modified Shepherd’s model charge and discharge voltage curves are based on Equation (2) and are represented below in Equation (3) and Equation (4).


$$V_{batt\_ch}(t)=E_{0}-K\frac{Q}{it-0.1Q}(it+i(t))+Ae^{-B\;it}-R_{o}i(t)\;(3)$$

$$V_{batt\_dis}(t)=E_{0}-K\frac{Q}{Q-it}(it+i(t))+Ae^{-B\;it}-R_{o}i(t)\;(4)$$

Where, *V _batt_ch_* represents the charge voltage and , *V _batt_dis_*) represents the discharge voltage. The rest of the parameters present in Equation (3) and Equation (4) ( *E*_0_), K, A, B) can be determined through the manufacturer discharge curve and being directly determined with the use of the equations of Table 1, shown below.

**Table 1.** Parameter’s description of equation 3 and equation 4.

Model parameter	Description	Equation representation
A, Exponential Voltage Amplitude Constant, in V	The amplitude of the exponential region.	*A* = *V*_*full*_ – *V*_*exp*_
B, Time Constant Inverse, in *Ah* ^–1^	Charge at the end of the exponential zone of the battery’s discharge curve. The scalar value of 2.3 was used in 31 to improve the fit to the specific battery used.	$B=\frac{2.3}{Q_{exp}}$
K, Polarization Constant, in *V/Ah*	Calculated using *V*_*full*_ and the end of the nominal zone of the discharge curve. The scalar value of 0.065 was used in 31.	*K* = *X*[ *V*_*full*_ – *V*_*nom*_ + *A*( *e* ^*–BQ*^_*nom*_) –1)] $\frac{Q_{full}-Q_{nom}}{Q_{nom}}$
R, Internal Resistance, in Ω	The internal resistance of the battery at steady-state current. *υ* is the efficiency of the battery, and *V*_*nom*_ and *Q*_*nom*_ are the nominal values for voltage (V) and energy capacity (Ah), respectively, of the curve (see Figure 2).	$R=V_{nom}\left(\frac{1-\upsilon }{0.2\times Q_{nom}}\right)$
*E*_0_, Battery Constant Voltage, in V	Represents the value when the battery is close to completely discharged and no current is flowing.	*E*_0_ = *V*_*full*_ + *K* + *R* × *i* – *A*

To calculate the voltage-current curve, the three points of the discharge curve are used (see Figure 2). In the work developed by the authors of 31, the proposed model considers this approach, with an inclusion of the battery lifetime. Moreover, the authors test the model within a SOC operating range in the linear region of the battery discharge curve (to operate within 20–85 % SOC range) and consider the temperature operating range to be maintained within the -10°C to 45°C range.

## 3. Methods

**Figure 3.**
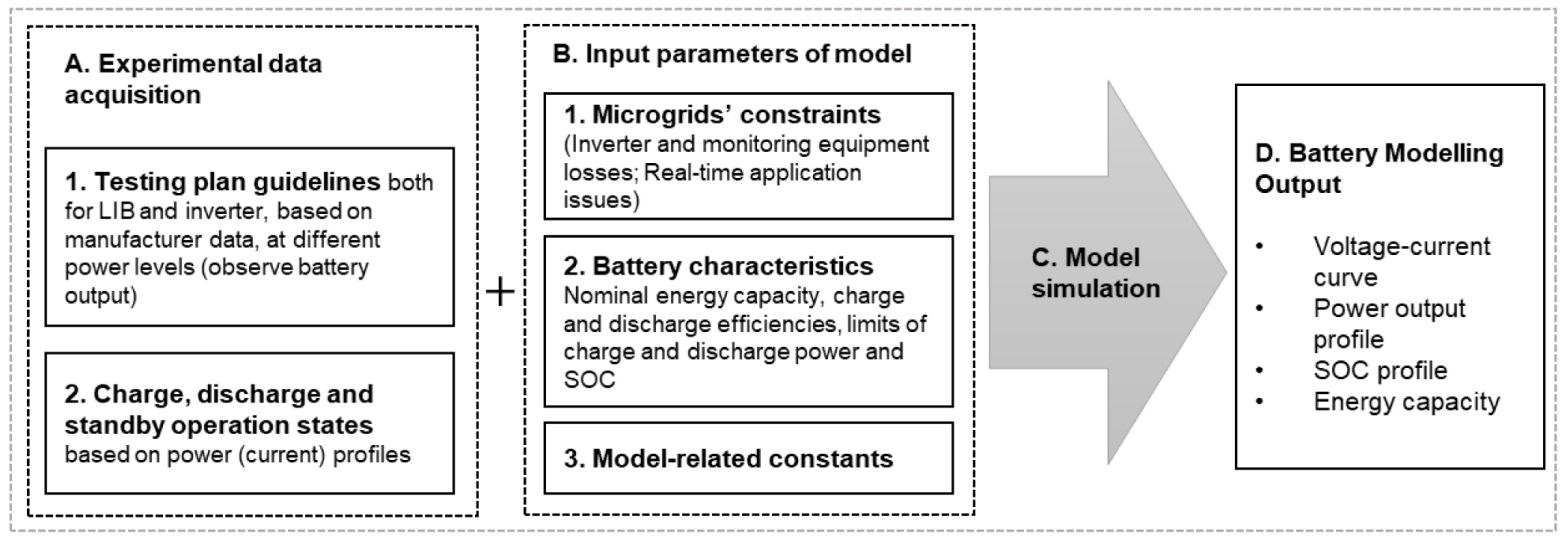
Methodology to describe the lithium-ion battery under study. LIB, lithium-ion battery; SOC, state of charge.

 In this work, the authors aimed to develop a model for a commercial LIB that allows its real-time description for stationary applications. An experimental setup is developed to characterize the battery, and further model validation against the experimental data acquired is carried out. The methodology follows the steps described in Figure 3, which details the input parameters subjacent to the model construction and development.

In the following subsections, the key aspects of the experimental data acquisition and the model approach are described.

### 3.1. University of Évora microgrid infrastructure

**Figure 4.**
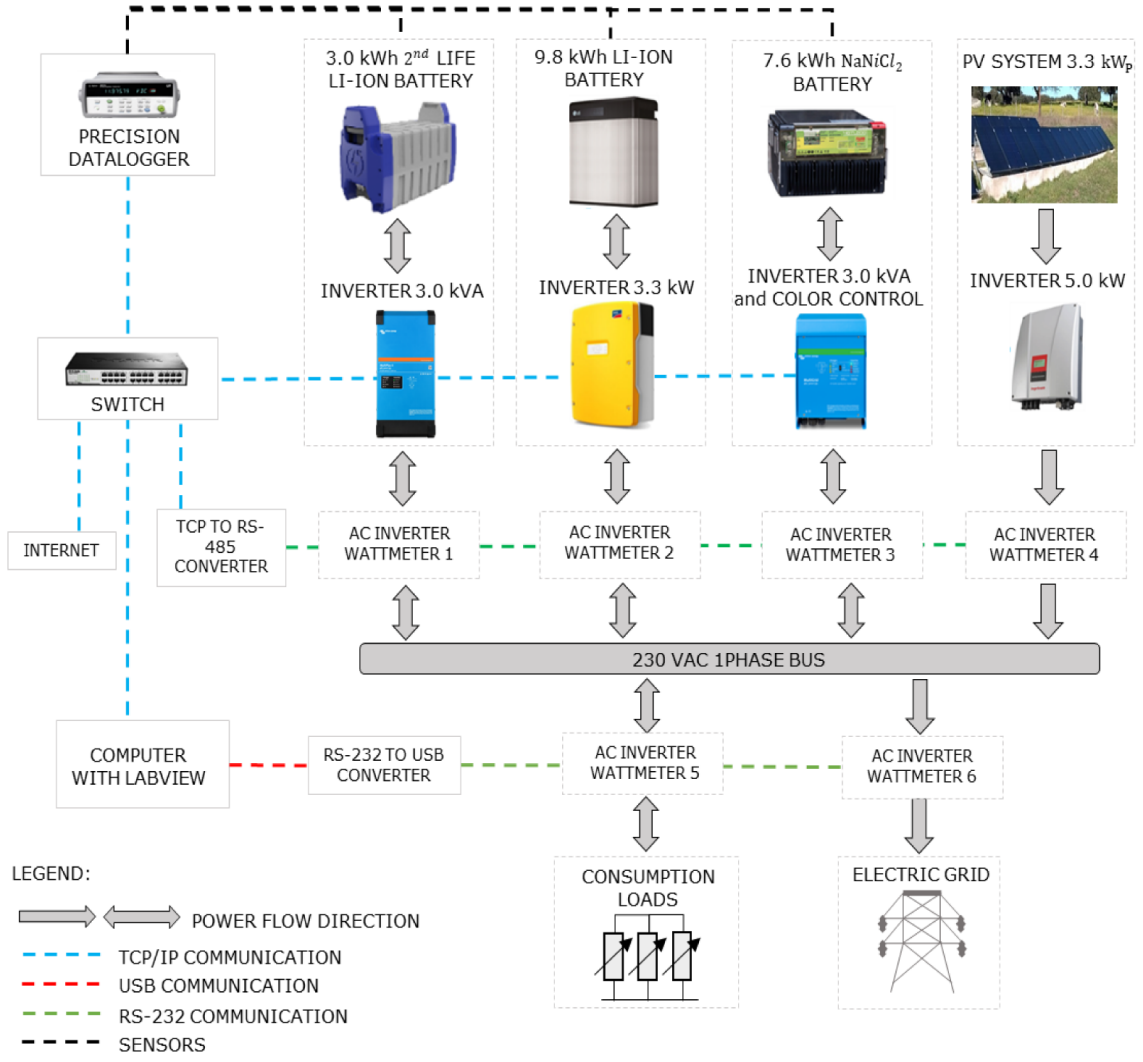
General schematic of the equipment and control platform of the microgrid of the Renewable Energies Chair at the University of Évora. In the figure, PV is the solar photovoltaic generation; AC is alternating current; TCP/IP is the Transmission Control Protocol/Internet Protocol (to allow communication on a network); and RS-232 is a protocol for the exchange of data (generally used in the serial ports of the computers).

The LIB characterization process aims at verifying the performance and dynamic behaviour of the selected battery: 5.0 kW/ 9.8 kWh (189 Ah) LIB energy storage from the LG Chem manufacturer ^36^, model RESU 10, with a nominal voltage of 48 V. The LIB and the SMA Sunny Island ^37^ 4.4M inverter (3.3 kW nominal power) are integrated into one of the University of Évora (UÉvora) microgrids. The integration consisted of connecting the systems to the grid, installing AC meters, DC measurement devices and outside temperature sensors (in the two surface parts of the battery and of the environmental temperature). The next step consisted in establishing a communication set with the inverter through the Modbus TCP/IP protocol (see Section 3.2 for further details). The current microgrid schematic is shown in Figure 4, including a 3.3kW solar amorphous photovoltaic installation, a 3.0 kW/ 7.6 kWh sodium-nickel chloride battery, and a 3.0 kW/ 3.0 kWh 2 ^nd^ life lithium-ion battery, and monitoring equipment (AC and DC meters and temperature sensors) on each relevant point of the microgrid. This research infrastructure was designed for the systems’ operation study and development, where a communication and control platform was developed. The manufacturer data ^38^ of the commercial battery is displayed in Table 2.

**Table 2.** Reference operating conditions and main characteristics available by the manufacturer of the LG Chem Resu10 48 V LIB ^38^.

Variable	Unit	Value
General		
DC voltage operating range	V	42.0~58.8
Volume (exterior box)	*m* ^3^	0.05
Weight	Kg	75
Average environmental temperature	°C	20
Depth of discharge	%	80
Energetic		
Nominal energy / useful	kWh	9.8 (25°C, 100% SOC) / 8.8
Nominal power / maximum / peak (in 3 s)	kW	3.3 / 5.0 / 7.0
Nominal Capacity	Ah	189
Maximum Current	A	119 at 42V
DC nominal Voltage	V	51.8
Cycles number (90% DOD, 25°C)	-	6000
Cycles number (80% DOD, 25°C)	-	10000
Environmental conditions		
Cooling	-	Natural convection
Operating Temperature	°C	-10 a 45
Optimal Operating Temperature	°C	15 a 30
Storage temperature	°C	-30 a 60
Humidity	%	5 a 95 (no condensation)
Altitude	m	< 2000

### 3.2. LIB and inverter testing description

In the consulted bibliography, battery testing procedures generally consist of a battery discharge at constant current, known as C-rate ^27, 31^. C-rate specifies the measuring rate at which a battery is discharged relative to its maximum capacity. In those references, the test consists of discharging the battery at a calibrated current. This fact led the battery modelling to be developed based on the manufacturer discharge curve at constant current, and this is one of the most widely models found in the literature ^27, 31, 39^. In the absence of both a manufacturer curve and the option of testing the LIB at a fully controlled constant current discharge (due to the inability to control all the inverter parameters, which satisfies the relation of power-voltage-current at each instant), the charge and discharge curves were obtained through the available points of control of the battery inverter, which is the alternating current (AC) delivery point.

In the following paragraphs, the experimental tests and respective data acquisition are presented. The outlined test plan considers the manufacturer's operating conditions (see Table 2), with a SOC range from 20–90%. The operation of the battery within the referred range includes a safety margin closely related to the depth of discharge (DOD) and the technology degradation. The environment temperature is controlled within the range of 15–25°C, achieved through the air-conditioning unit. To be able to carry out the testing plan and real-time data monitoring, a battery-inverter communication control was developed, based on the Modbus TCP/IP protocol ^40, 41^, with the help of the LabVIEW 2014 programming environment. Modbus protocol interface addresses’ map is made available by the inverter manufacturer SMA Sunny Island 4.4M ^41^. The code developed in LabVIEW is also attainable through open-source software e.g. Python, using the Modbus TCP/IP communication protocol and the manufacturer map.

Through this communication, the inverter is asked to, periodically (at 2-second intervals), retrieve or send commands (based on the power/current profiles at the AC point connection). The data acquisition is achieved by reading the parameters of the inverter, from the high precision AC wattmeter ^42^, and from the measuring precision datalogger ^43^. The acquired parameters include current, voltage, power, temperatures, and battery and inverter alarms.

**Figure 5.**
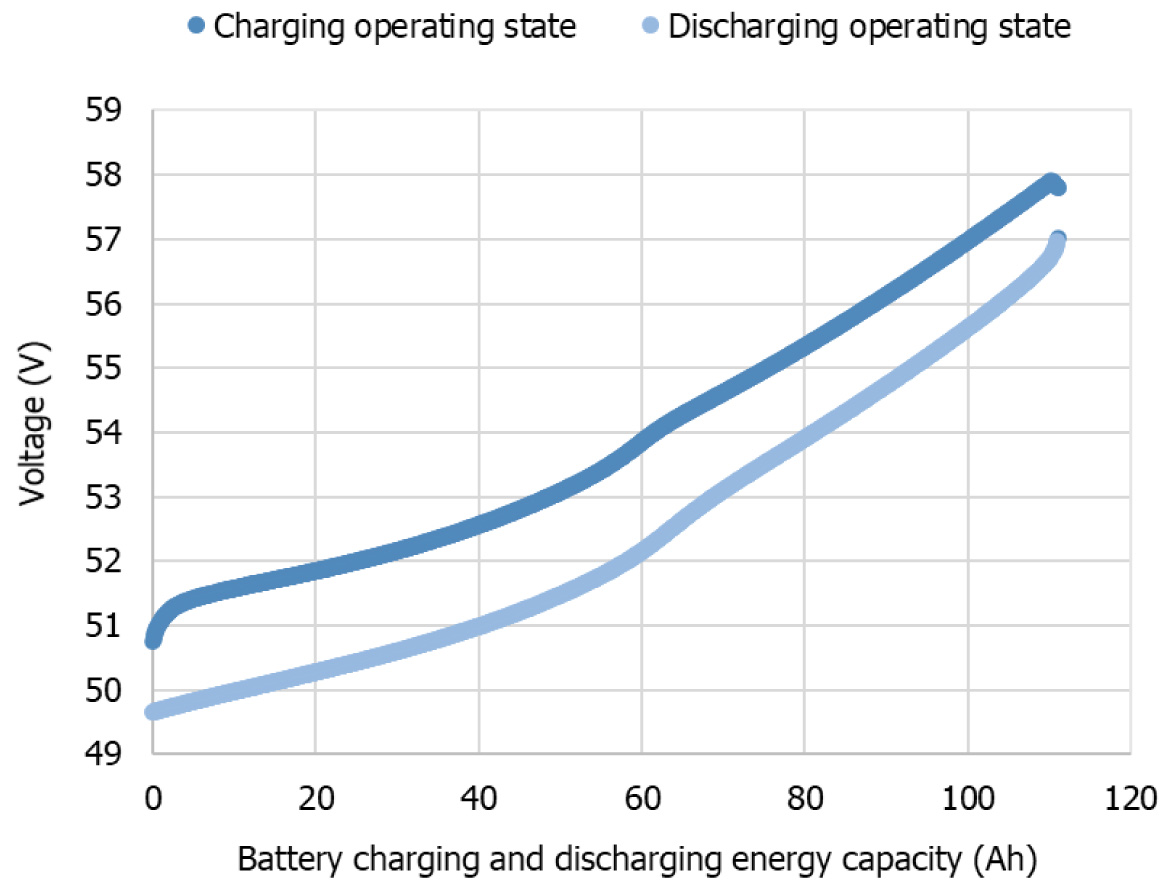
Complete battery charge-discharge cycle at a constant power level of 2.7 kW at the alternating current delivery point, from 20–90 % state of charge range.

After assuring reliable data measurement acquisition and optimization of the control program, battery cycling tests were conducted. The cycling corresponded to 30 complete charge-discharge cycles at predefined constant power levels throughout the SOC range (from nominal power to low operating power). The obtained average data for each of the power levels is made available in *Underlying data* ^44^. An example of a full charge and discharge battery test is shown in Figure 5, at a constant power level of 2.7 kW, throughout the mentioned SOC range.

Given the integrated battery-inverter use, it is important to consider the efficiency curve of the inverter, which varies throughout its power range (either charging or discharging). For that reason, it is relevant to acquire the experimental efficiency curves since it will impact the overall battery system results. The inverters or power converters operate at different power levels and according to a power-related efficiency profile.

To evaluate the performance of the SMA Sunny Island 4.4M inverter, a test control was implemented in the LabVIEW 2014 programming interface, fully reproducible through open-source software, e.g., Python, using Modbus TCP/IP and achieved through the mapping of addresses given by the inverter manufacturer (in this case, SMA) ^41^. The test control made was similar to the previous, where only the time of remaining at a certain level of power was variable. The protocol consisted in the operation of the inverter at distinct rates of its nominal power (5% to 100% rate), during periods of 15 minutes. DC and AC electrical parameters were monitored. The main objective of the test was the calculation of the charge and discharge efficiencies over the power operating levels, presented in the next section of this work ( Section 4).

### 3.3. LIB modelling application

Taking advantage of the previously obtained experimental data to describe the LIB, a LIB model was developed, considering modelling adaptations of Shepherd’s model, described in Section 2.2.3. In the work developed in 31, the authors describe the battery through the manufacturer-provided curves. In the present work, the battery is described by the results of the experimental testing at different constant power levels over the complete charging and discharging operating states. The modified version of Shepherd's model was computed to obtain the battery dynamic behaviour, using Equation (3) and Equation (4), considering the dynamic current over the state of charge range, at a certain level of power.

In addition, the state of charge parameter of the battery at each instant *t* is generally estimated by the following Equation (5) and Equation (6),


$$SOC(t)=SOC(t-1)+\frac{i(t)\times \text{Δ}t}{Q}\;,\;\text{if}\;\text{Q}\;\text{is}\;\text{in}\;\text{the}\;\text{unit}\;\text{of}\;\text{Ah}\;(5)$$

$$SOC(t)=SOC(t-1)+\frac{P(t)\times \text{Δ}t}{Q}\;,\;\text{if}\;\text{Q}\;\text{is}\;\text{in}\;\text{the}\;\text{unit}\;\text{of}\;\text{Wh}\;(6)$$

Where Q is the energy capacity of the battery, and the Δ *t* the difference of the step time: *t* – 1 (previous instant of *t*) and *t*. The values of *Q* are bounded by its real defined range of minimum and maximum energy capacity values. Taking the model of Section 2.2.3. and to better describe the LIB under study, some adaptations were made. In Equation (3), a multiplication factor of 0.65 for Q was used, instead of the 0.1 described. Regarding the rest of the parameters, the used time constant inverse of 2.3/ *Q _exp_ Ah* ^–1^ was maintained, and a polarization constant with a scalar value of 7.3 was used instead of 0.065 (see Table 1). For the obtained voltage to be described by Equation (3) and Equation (4), the curve has a constant fit of a scalar value of +41.2.

The model was computed using MATLAB (2017b) programming and is fully reproducible through alternative software, e.g. Python (see *Software availability* ^45^). As a first approach, the operation of the battery at constant power levels, acquired experimentally, was chosen to validate it. From the experimental values, the model was used to suit both charge and discharge curves, given the need to describe the overall battery behaviour. For the intermediate power values, a technique of regression fittings was used, taking advantage of the MATLAB fitting tool (also existing in alternative software, as the open-sourced software Python).

To represent the LIB voltage behaviour, the proposed model uses the current as input. Regarding the practical model application within a larger model regarding all microgrid systems (and to implement energy management strategies), it is generally useful to have the battery output in terms of the energy and power domains. The cp-rate can be defined by the rate of constant power that will cause the battery to discharge in a certain amount of time, as explained in 31.

***3.3.1. LIB ageing model.*** The battery lifetime depends on the operation conditions based on temperature, SOC and total energy throughput (electrochemical operating ranges), charge and discharge rates ^46^ and the total number of cycles. In the case of the present work, particular emphasis will be given in the description of the battery in its current state, through the validation of the model approach against the experimental results. The main goal is to have the battery model in its current operational state, with validation of this model approach with the experimental results. Nevertheless, the LIB ageing effects should be included in the modelling used for real-time predictive optimization control. There is no common model for LIB ageing, although this model should describe the fade mechanisms, triggered by cycling patterns, storage, and others. Lifetime fade or degradation (capacity decrease) and cycling fade affect the performance and battery lifetime, having a real impact on its economic results (given the high upfront cost).

**Figure 6.**
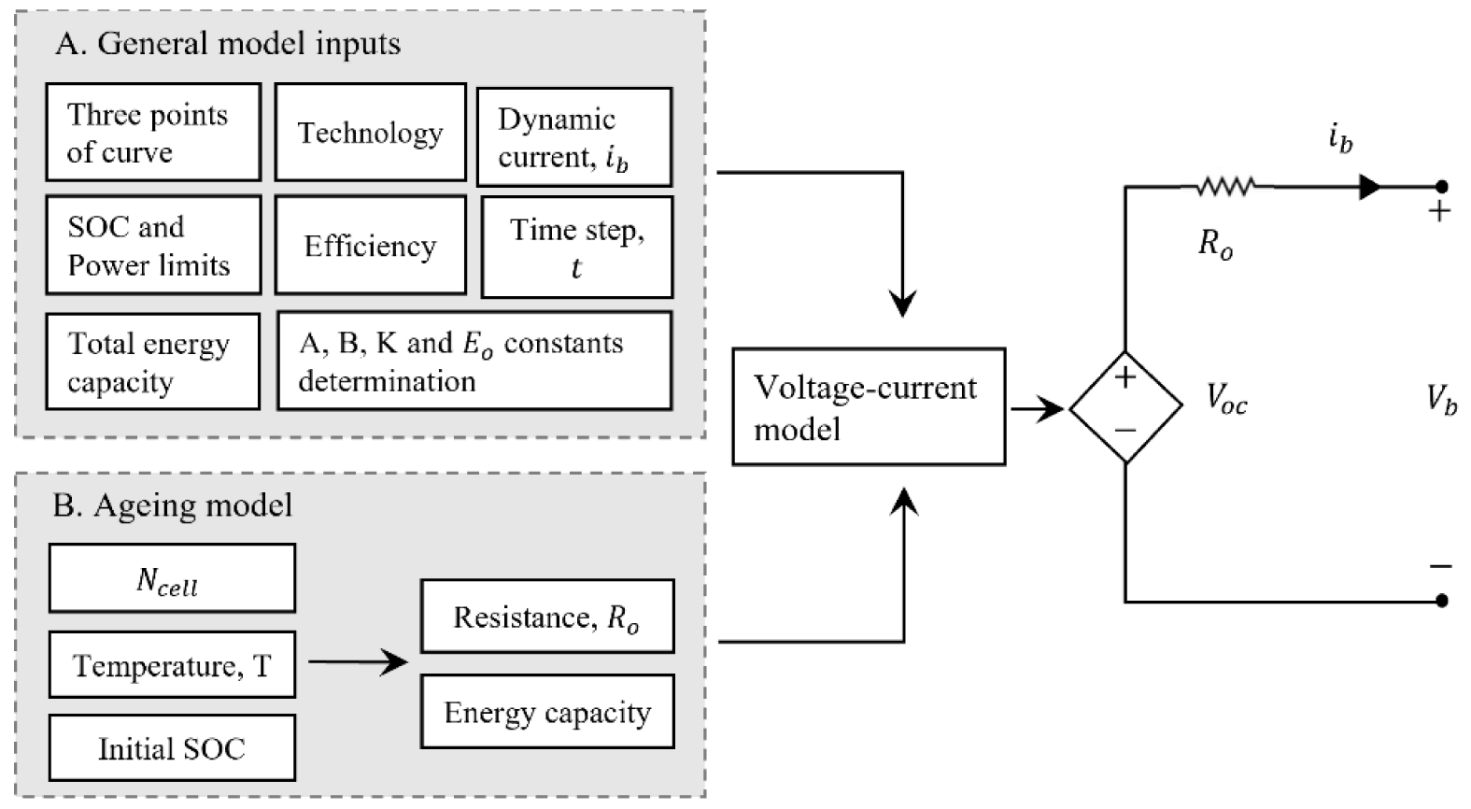
Complete model of the lithium-ion battery technology approach. In this figure, A is the exponential voltage amplitude constant (V); B is the time constant inverse (Ah); K is the polarization constant (V/Ah); *E*_0_ is the battery constant voltage (V); *N _cell_* the number of cells in series; SOC is the state of charge of the battery; *R*_0_ is the series resistance of the equivalent model (Ω); *V _oc_* is the open circuit voltage (V) and *V _b_* is the battery terminals voltage (V).

Given the present work approach, it is possible to update the values of internal resistance (depending on temperature and state of charge) and energy capacity (depending on temperature, T and cycle count, *N _cell_*), based on the National Renewable Energy Laboratory (NREL) lifetime model ^46^, which will be included in detail in further work application of this model. The final complete LIB long-term use model is shown in Figure 6.

## 4. Results

This section summarizes the main output results of this work, firstly concerning the battery pack and inverter characterization performance, and secondly, the developed model which fit the experimental data previously obtained.

### 4.1. Battery testing and inverter results

Based on the methodology described in Section 3, the battery and inverter characterization data were obtained, and the battery performance indicators were calculated (see *Underlying data* ^44^). The characterization included the realization of at least three cycles for each power level, to obtain greater precision and accuracy within the results.

**Figure 7.**
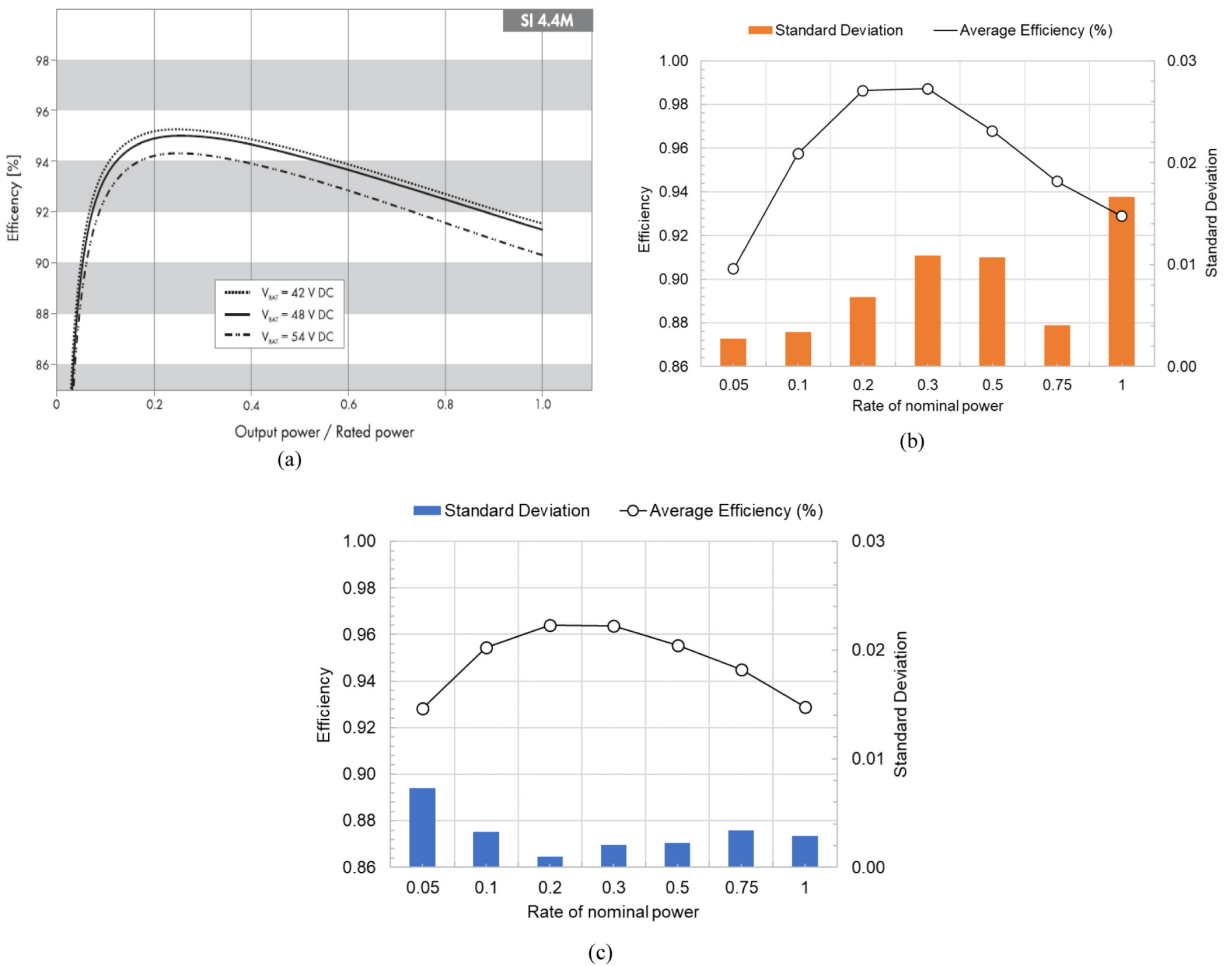
Inverter efficiency curve as a function of the power level ( **a**) given by the manufacturer ^47^, ( **b**) from the experimental results of discharge, and ( **c**) from the experimental results of charge. In this figure, ( **a**) has been reproduced with permission from 47.

Concerning the inverter charge efficiency, it was possible to obtain an average value of 94.8 %, with an STD of 1.4, and regarding discharge, the obtained average value was 95.6 % with an STD of 2.7. The efficiencies defined by the manufacturer are an IEC efficiency of 94.0 % and a maximum efficiency of 95.3 %. Figure 7 presents the data from the inverter manufacturer in the technical document (a) ^47^ and the experimentally data obtained for different power levels for discharge (b) and charge (c).

Regarding the battery, the calculation of the charge and discharge efficiencies was based on the DC measurements acquired throughout the tests, presented in Table 3.

**Table 3.** Charge and discharge battery cycling results, at constant values of power over the state of charge range.

Rate of nominal power	AC Power (W)	DC Energy	
		Average efficiency	Standard deviation
10	330	0.75	0.05
15	500	0.84	0.01
20	650	0.85	0.00
30	1000	0.84	0.07
50	1650	0.90	0.03
60	2000	0.95	0.10
75	2500	0.96	0.05
80	2700	0.79	0.00
100	3300	0.74	0.01

**Figure 8.**
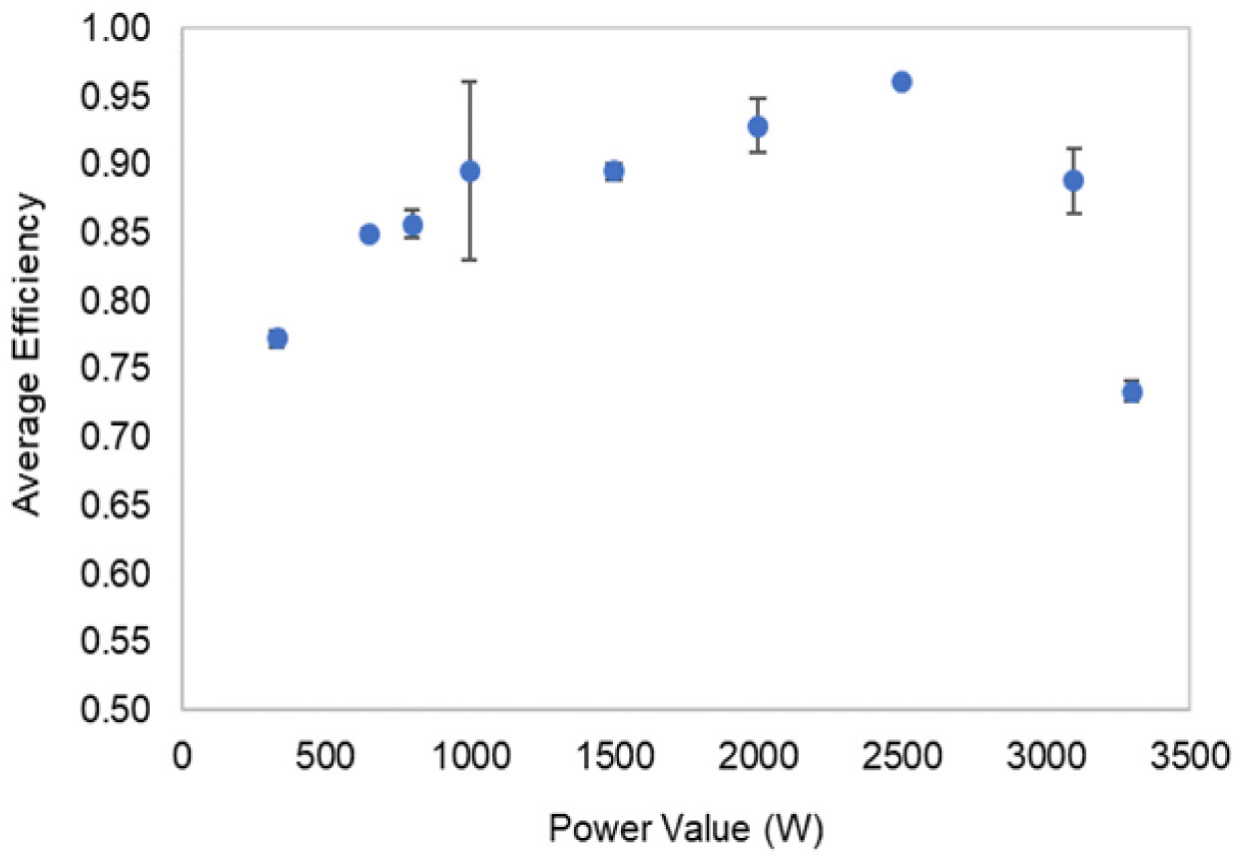
Battery efficiency at each level of power, and respective standard deviation results.

Figure 8 presents the battery average efficiency in each power level and the correspondent STD. From the experimental results, it was possible to obtain an overall average battery efficiency of 84.6 % with a STD of 7. In Figure 8, the influence of the inverter efficiency dependency on power rating is noticeable.

The accomplishment of these testing’s allows the calculation of other battery indicators besides efficiency, as energy capacities, energy densities, full charge and discharge spent times, response times, and typical discharge power. These indicators are presented in Table 4.

**Table 4.** Battery performance indicators resulting from the experimental tests.

Battery Performance Parameters	Units	Results
Total Capacity (Charge capacity)	kWh	6.6 ± 0.5
Useful maximum capacity (Discharge capacity)	kWh	5.5 ± 0.4
Energy Density in charge by unit of mass	Wh/kg	86 ± 6.0
Energy Density in discharge by unit of mass	Wh/kg	73 ± 5.0
Energy Density in charge by unit of volume	Wh/L	129 ± 10
Energy Density in discharge by unit of volume	Wh/L	109 ± 8.0
Fastest/slowest charge	h	2h04min / 10h46min
Fastest/slowest discharge	h	1h33min / 3h48min
Charge/discharge efficiency (complete cycles)	%	84.6 ± 0.1
Charge/discharge maximum power	kW	3.3
Response Time	Seconds	< 1s
Typical discharge time	h	Hours

**Figure 9.**
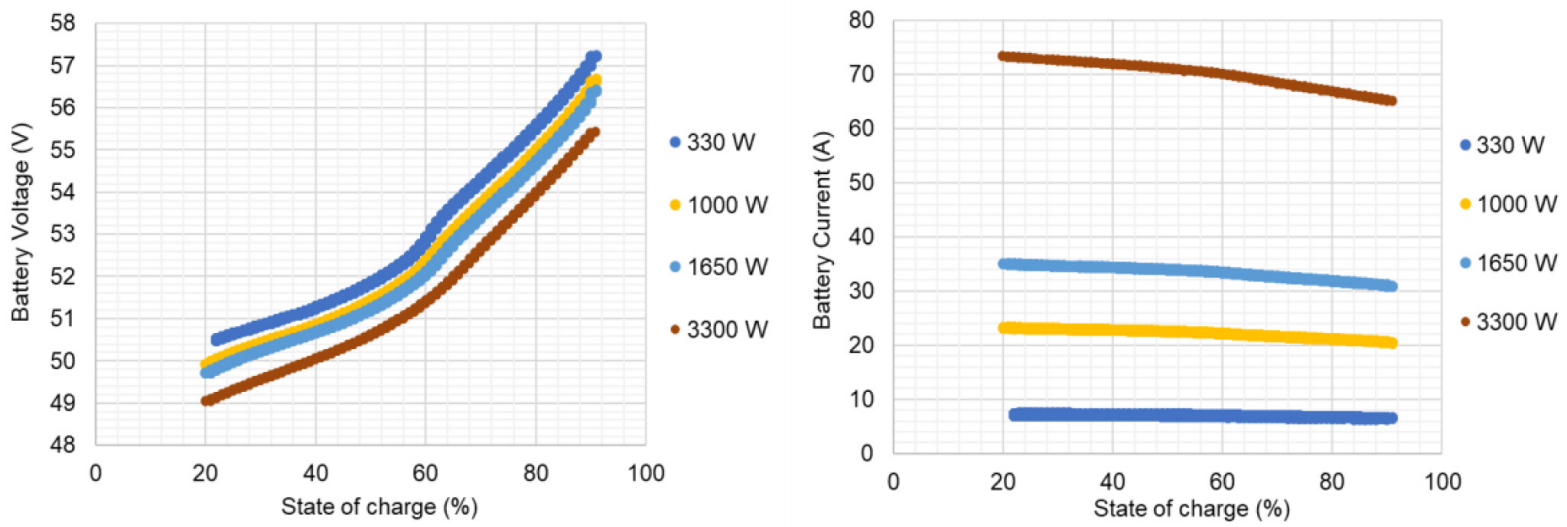
Lithium-ion battery voltage and current data from the experimental test plan, for complete charge-discharge cycles, at different constant power levels (due to readiness, only few power levels are represented).

The experimental acquired voltage-current data in function of the battery energy capacity (or SOC) were used to validate the modelling approach of the next Subsection (4.2). Figure 9 exemplifies some of the experimental results obtained.

### 4.2. Modelling approach to the overall battery operating states

In the literature is common to find references on the battery modelling for one c-rate ^27, 31^, but it lacks a solution to describe the battery behaviour over its overall operating frame, required to meet the aim of this work. The validation of a single power level charge and discharge curve, chosen as the nominal power, was conducted. Having tested the battery at distinct power levels and considering the validated data for one power level, it was possible to find a regression fit for the remaining power levels, based on the variation of the three extracted points from the curves but keeping all the other variables as constant. Below, the approach is detailed.

***4.2.1. Battery behaviour representation for a single power level.*** For the nominal inverter power level, 3300 W, over the defined SOC range, and at a constant ambient temperature of 20°C (measured), the voltage-curve was profiled and used for later model validation. For both experimental curves of charge and discharge – on the contrary of the manufacturer curves as the modified Shepherd’s model defined – the three extracted points of voltage and capacity are hereinafter enunciated, for the charge in Equation (7),


$$\;\{\begin{array}{c} \left(V_{full},Q_{full})=(60.90,169\right)\\ \left(V_{exp},Q_{exp})=(57.35,Q_{full}\times n_{\exp\_c}\right)\\ \left(V_{nom},Q_{nom})=(52.60,Q_{full}\times n_{nom}\right) \end{array}\;(7)$$

And discharge, in Equation (8),


$$\;\{\begin{array}{c} \left(V_{full},Q_{full})=(60.90,169\right)\\ \left(V_{exp},Q_{exp})=(58.50,Q_{full}\times n_{\exp\_d}\right)\\ \left(V_{nom},Q_{nom})=(54.60,Q_{full}\times n_{nom}\right) \end{array}\;(8)$$

Where *n*_exp_ *c*_ is a percentage of the *Q _full_* to obtain the value of *Q _exp_* in the case of charge, and *n*_exp_ *d*_ in the case of discharge. The *n _nom_* is a percentage of the *Q _full_* to obtain the value of *Q _nom_*. In the case of the discharge state, the same percentage is given both for charge and discharge.

**Figure 10.**
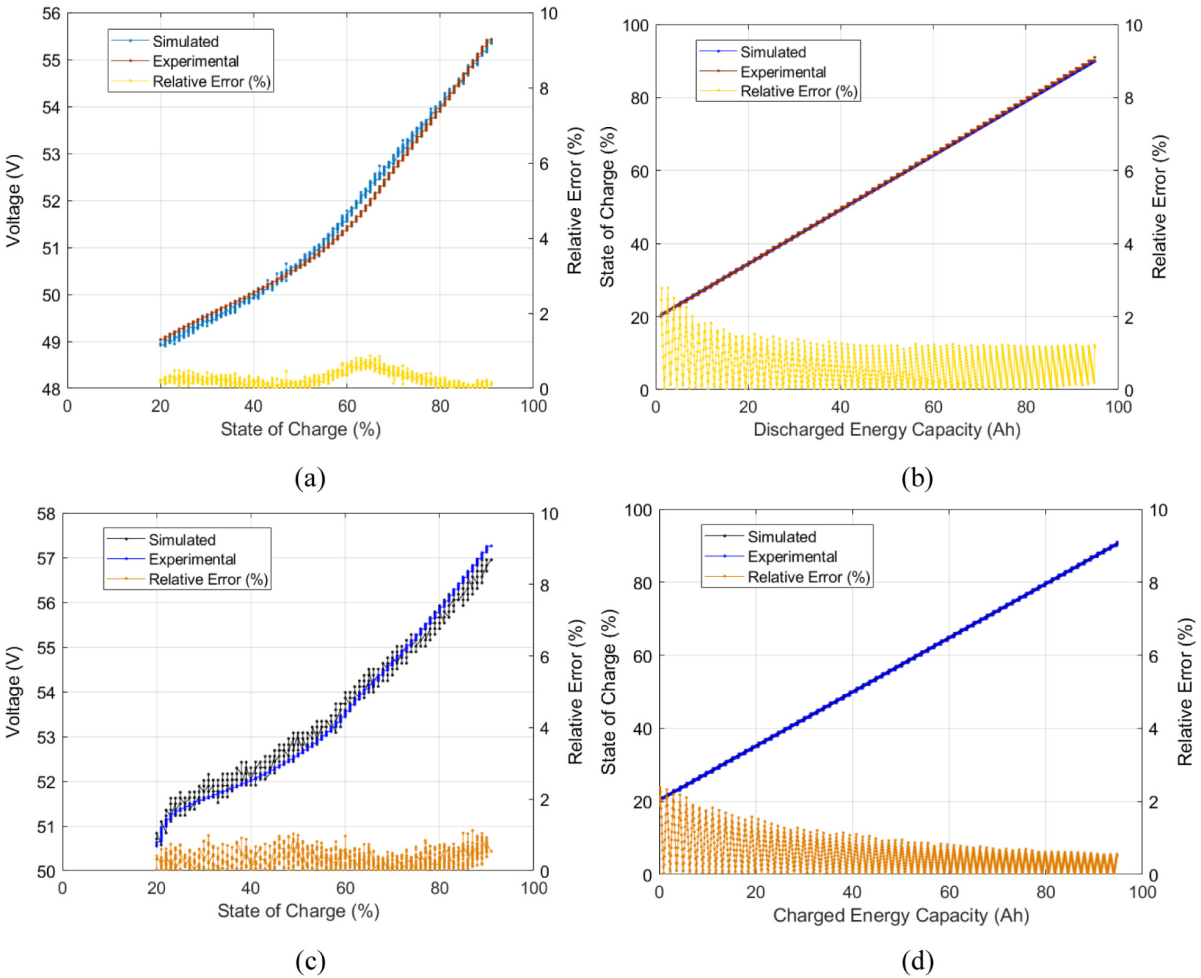
Model validation simulated vs experimental results for the 3300 W level of power: ( **a**) discharge voltage- State of Charge (SOC) curve, ( **b**) discharge SOC-energy capacity curve, ( **c**) charge voltage-SOC curve, and ( **d**) charge SOC-energy capacity curve.

The charge and discharge voltage curve for this power level presented a root mean square error (RMSE) of near 0.1 V and a maximum relative error of near 1.0 %. The obtained SOC values presented a maximum relative error of near 2.5 %. The experimental and simulated voltage curves for this power level are shown in Figure 10.

***4.2.2. Battery behaviour representation for all power levels (regression fit).*** Figure 10 offers the observation of the battery voltage curve and over the different states of charge, and SOC vs. energy capacity of a single power level. To have an overall battery description, distinct experimental power level curves should be tested within the simulated model. For the model to describe the different operating power levels of the battery, the three points of voltage and energy capacity (see Equation (7), Equation (8) and Figure 2) that correspond to the experimental charge and discharge curves of each power level (or different current level), should be extracted (guessed). To that sense, the following approach aims to adjust the simulated model curves of each power level to the experimental results obtained, as Equation (9) indicates,


$$\;\{\begin{array}{c} {\left(V_{full},Q_{full}\right)}_{power\_level(soc)}=(V_{full}+Y,Q_{full}+Z)\\ {\left(V_{exp},Q_{exp}\right)}_{power\_level(soc)}=(V_{exp}+Y,Q_{exp}+Z)\\ {\left(V_{nom},Q_{nom}\right)}_{{power}_{level(soc)}}=(V_{nom}+Y,Q_{nom}+Z) \end{array}\;(9)$$

**Figure 11.**
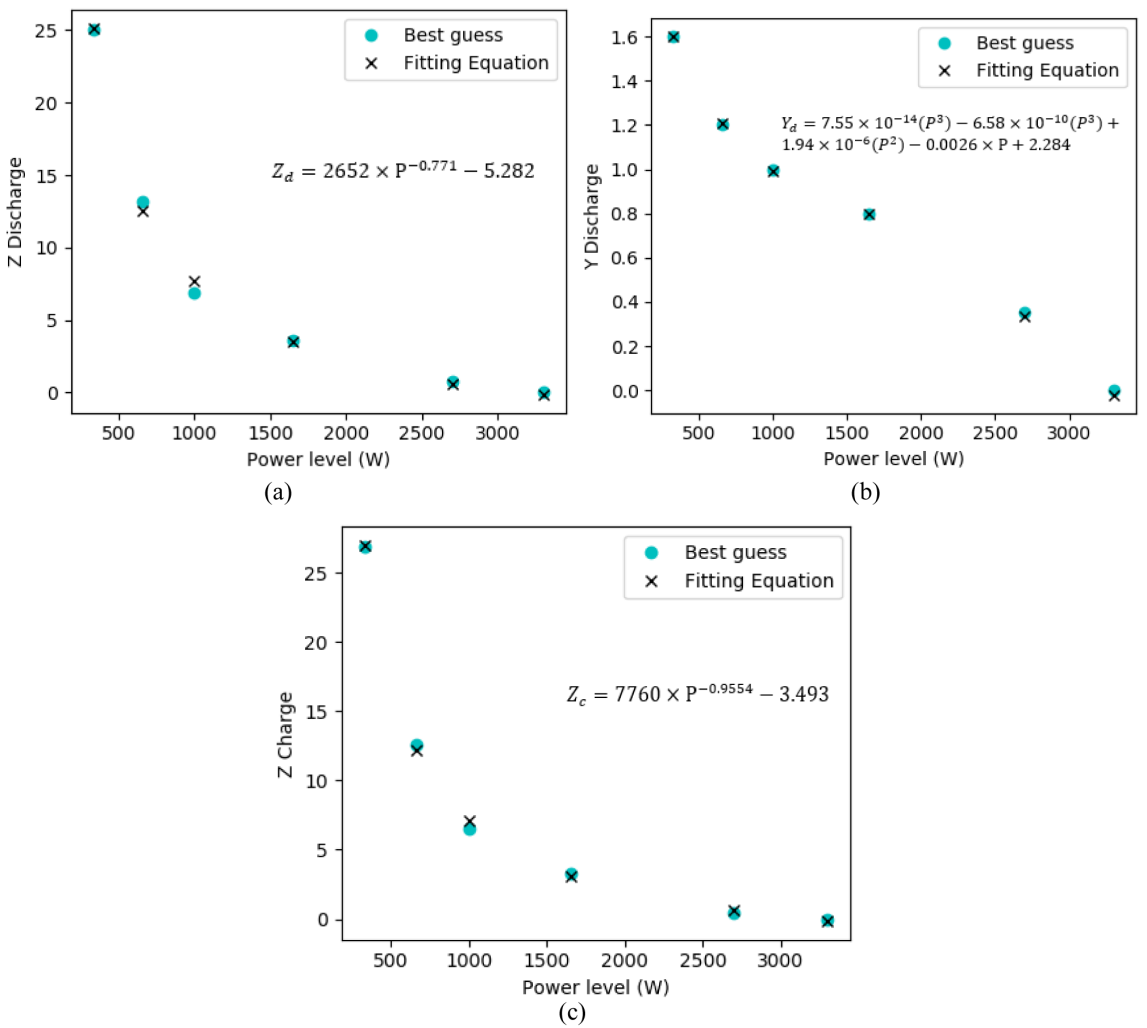
Y and Z regression fitting values obtained for the charge and discharge operating states, in function of the power levels of the battery operation. In these figures, the guessed values that better described the three extracted points were put against the best-fit function.

Equation (9) was used to check the existence of a relationship of Y and Z with the extracted points (guessed) that better describe each power level experimental values. As firstly made to the first power level ( Section 4.2.1), points from the experimental curves of each power level were guessed, and afterwards, the values of Y and Z that better fit that data are obtained. A curve-fitting on the best-guessed values which described the experimental data of the battery was made, with the help of the MATLAB curve fitting tools (also existing in alternative software’s, as the open-sourced Python), both for charge and discharge operation state. Through the curve fitting, it was possible to find a function that describes the values of Y and Z that should be added/subtracted to the initial guessed three points of the curves. The functions’ representations are shown in Figure 11.

The determination of Y and Z for both charge and discharge states offer, in this way, a full battery behaviour description within the experimental values obtained. To observe the voltage regression fit accuracy of the simulated values against the experimental acquired values, Table 5, and Table 6 present the RMSE and maximum relative errors of each fitting point with its correspondent experimental value for discharge and charge operating states, respectively. The obtained regressions allow the description of the battery through the distinct operation states of the battery in the regular operating conditions. The state of charge at each of the power level obtained values can be observed in Table 7. The developed model is made available in *Software availability* ^45^.

**Table 5.** Comparison of the simulated against experimental voltage values on discharge state, and respective errors (root mean square error (RMSE) and minimum and maximum relative errors). Where, *n*_exp_ *d*_ is a percentage of the *Q*_*full*_ to obtain the value of *Q*_*exp*_ in the case of discharge curve; *n*_nom_ is a percentage of the *Q*_*full*_ to obtain the value of *Q*_*nom*_; *Y*_*d*_ and *Z*_*d*_ are the values to be added to the voltage and energy capacity, respectively, extracted discharged curves.

Power level (W)	*n*_exp_ *d*_	*n*_*nom*_	*Y*_*d*_	*Z*_*d*_	RMSE error (V)	Maximum Relative Error (%)
330	0.77	0.98	1.60	25.1	0.74	4.81
660			1.21	12.5	5.51	5.68
1000			0.99	7.65	1.28	3.11
1650			0.80	3.51	0.48	2.12
2700			0.35	0.73	0.05	1.17
3300			0.01	0.13	3.84	4.78

**Table 6.** Comparison of the simulated against experimental voltage values on charge state, and respective errors (root mean square error (RMSE) and minimum and maximum relative errors). Where, *n*_exp_ *c*_ is a percentage of the *Q*_*full*_ to obtain the value of *Q*_*exp*_ in the case of charge curve; *n*_nom_ is a percentage of the *Q*_*full*_ to obtain the value of *Q*_*nom*_; *Y*_*c*_ and *Z*_*c*_ are the values to be added to the voltage and energy capacity, respectively, extracted charged curves.

Power level (W)	*n*_exp_ *c*_	*n*_*nom*_	*Y*_*c*_	*Z*_*c*_	RMSE error (V)	Maximum Relative Error (%)
330	0.60	0.98	0.00	26.9	1.34	5.57
660			0.00	12.2	5.27	6.74
1000			0.00	7.07	1.06	2.75
1650			0.00	3.05	1.02	3.02
2700			0.00	0.60	0.47	3.78
3300			0.00	0.12	0.10	1.61

**Table 7.** Comparison of the simulated against the experimental State of Charge (SOC) values errors (root mean square error (RMSE) and minimum and maximum relative errors).

Operating state	Charge		Discharge	
Power level (W)	RMSE error (V)	Maximum Relative Error (%)	RMSE error (%)	Maximum Relative Error (%)
330	0.78	3.12	0.60	3.90
660	0.37	2.97	0.25	3.67
1000	0.30	6.53	0.31	3.60
1650	1.15	2.61	0.48	5.02
2700	0.20	2.62	0.79	6.02
3300	1.05	2.44	0.30	6.82

## 5. Discussion

In this work, a commercial LIB battery pack was the object of experimental tests, through its complete charge and discharge at constant levels of a power testing plan. The manufacturer data helped to track the operating range limits and, in the testing, the overall conditions definition. The establishment of inverter communication allowed testing of the battery under controlled conditions. For that reason, the testing output is influenced by the inverter unit. The LIB testing results, which enabled the energetic performance calculation presented in Table 4 and Figure 8, are in accordance with the expected results for the technology in test ^48, 49^. In addition, the values obtained for the inverter efficiencies are close to the manufacturer-provided data, with a calculated STD of 2.7 %.

A LIB model is commonly found in literature, mostly based on the MATLAB/Simulink pre-existing model ^27, 29, 30, 50^. The reproduction/justification of the model to be applied for a stationary application which could fit the obtained experimental data was not found. Thus, in this work, the authors based the modelling approach on one of the modified Shepherd’s models and adapted it to describe the real-time battery dynamic behaviour of the battery integrated into the microgrid.

The results obtained within the model approach used show satisfactory adequacies for all the values experimentally obtained with the LIB. In discharge state, the higher voltage RMSE is 5.51 V and a maximum relative error (MRE) of 5.68 %, regarding SOC the MRE obtained was about 6.82 %. In charge state, the highest RMSE voltage is 5.27 V, a MRE of 6.74 % and concerning SOC the MRE obtained is about 6.53 %. Given the equation fitting and the experimental data errors obtained, generally, the model describes the battery behaviour. The standby operation (self-discharge and inverter auxiliary consumption) will also be considered in future work for the final complete model.

The temperature effects were not fully considered in this approach. The decision was based on dependence variation within the series resistance, which impacts battery lifetime. This question will be approached in further research considering the model describing the battery behaviour over time (to be used in future energy and economic assessments) and including temperature and SOC effects in the resistance calculation, as well as the temperature and cycling effects in the calculation of battery capacity.

The achieved model describes with small computational effort and fast runtime the main characteristics of the battery, which facilitates its application within larger models of energy management strategies. The model is validated with different power levels experimental battery data, improving its modelling of the battery behaviour with variable input profiles.

Similarly, for most commercially available LIBs, the LIB manufacturer does not provide information regarding the internal construction, missing information such as the number of cells, parallel and series connections, voltage equalization algorithms, temperature control algorithms, etc. This missing information limits the modelling accuracy, generating a higher error in the simulation results. Nevertheless, the maximum error obtained is considered low, indicating the model accuracy for describing the battery performance, even with important battery data not being provided by the manufacturer.

## 6. Conclusions

This work applies and validates a model to a 9.80 kWh (189 Ah) lithium-ion commercial battery pack behaviour – voltage-current curves, energy capacity and SOC profiles with real-time variation – to give a potential modelling application to optimization and predictive microgrid programming control (including additional assets and corresponding models, such as PV systems), specifically for commercial and residential applications.

The correspondence between the general voltage-current models and the operating conditions matching of the battery is usually a complex task. In this work, a LIB and inverter experimental setup was built for the characterization of performance and behaviour with precision monitoring. Communication was established with the battery inverter, enabling it to send real-time commands and get readings. This setup allowed us to perform the necessary characterization tests under real operating conditions.

Several curve fittings that represent with low error the battery behaviour under different operating states were obtained. Charge and discharge errors were calculated and can be observed in Table 5, Table 6, and Table 7, with a maximum relative error of 6.74 % for simulated voltage.

Future development of this work will include the application of this model within a larger simulation model, considering additional systems present in the residential and commercial sectors. Further development should be made to also include an ageing model and an energy management strategy, giving its importance to a technical-economical evaluation result. Lithium-ion battery technology will continue to increase within its expected application in the automotive market, with the expectation that the technology will also be used in a second market for stationary applications. Accurate models for stationary application of LIB will provide important advantages to the market uptake, in particular, for the residential and commercial sectors.

## Data availability

### Underlying data

Zenodo: Lithium-ion battery charge and discharge testing data - current, voltage, soc, ta - at constant levels of power. https://doi.org/10.5281/zenodo.5196334 ^44^.

This project contains the following underlying data:

– Lithium_Ion_Battery_Testing_Data.csv (this dataset was used in the composition of the lithium-ion battery testing and modelling validation. The file contains the charge and discharge testing acquisition data - current, voltage, soc, ta - at constant levels of power. The legend of the text is given in the final column “BI” of the .csv file.)

Data are available under the terms of the Creative Commons Attribution 4.0 International license (CC-BY 4.0).

## Software availability

Source code available from: https://github.com/catSelof/Batteries

Archived source code at time of publication: https://doi.org/10.5281/zenodo.5814884 ^45^

License: LGPL-2.1

## Ethics and consent

Ethical approval and consent were not required.
