# Validation of a lithium-ion commercial battery pack model using experimental data for stationary energy management application

**DOI:** 10.12688/openreseurope.14301.2

**Published:** 2022-06-10

**Authors:** Ana Foles, Luís Fialho, Pedro Horta, Manuel Collares-Pereira

**Affiliations:** 1 Renewable Energies Chair, University of Évora, Pólo da Mitra da Universidade de Évora, Edifício Ário Lobo de Azevedo, 7000-083 Nossa Senhora da Tourega, Portugal; 2 Institute of Earth Sciences, University of Évora, Rua Romão Ramalho, Évora, 7002-554, Portugal

**Keywords:** Electrical energy storage, lithium-ion battery, characterization tests, battery model

## Abstract

**Background: **A cost-effective solution for the design of distributed energy storage systems implies the development of battery performance models yielding a suitable representation of its dynamic behaviour under realistic operation conditions.

**Methods: **In this work, a lithium-ion battery (LIB) is tested to be further modelled and integrated into an existing energy management control system. This specific LIB (5.0 kW /9.8 kWh) is integrated with a commercial inverter and solar photovoltaic (PV) system (3.3 kWp) as part of a microgrid that is also encompassing other energy storage technologies at the University of Évora, Pole of INIESC – National Research Infrastructure for Solar Energy Concentration. A testing protocol fully characterizes the battery and the inverter efficiency to describe their performance better. Then, a battery model is built upon both the existent LIB description and experimental fitting regression. The model allows obtaining the voltage curve, the internal resistance (i.e., to describe instantaneous voltage drop/rise and transients), and the state of charge (SOC) and/or energy capacity based on the current input. The developed model is validated through the comparison with the experimental results.

**Results: **The model approach presented a higher voltage RMSE (root mean square error) of 5.51 V and an MRE (maximum relative error) of 5.68 % in the discharge state. Regarding SOC, the MRE obtained was approximately 6.82 %. In the charge state, the highest RMSE voltage was 5.27 V, with an MRE of 6.74 %. Concerning SOC, the MRE obtained was approximately 6.53 %.

**Conclusions: **The developed model is validated through the comparison with experimental results. Based on computational effort, simplicity of use and the associated model error, the approach is validated to the regular conditions of the commercial battery pack to be incorporated in the next research step, following a bottom-up modelling approach for an increasingly more complex smart grid.

## Nomenclature

**Table:** 

*Abbreviation*	*Definition*
BESS	Battery Energy Storage Systems
BMS	Battery Management System
DOD	Depth of Discharge
DSM	Demand Side Management
DSO	Distributor System Operator
ECM	Electric Circuit Model
EES	Electrical Energy Storage
EMS	Energy Management Strategy
ESS	Energy Storage System
EV	Electric Vehicle
LIB	Lithium-ion Battery
R&D	Research and Development
RES	Renewable Energy Sources
SOC	State of Charge (%)
TSO	Transmission System Operator
VRE	Variable Renewable Energy

## 1. Introduction

In 2019, 2.9 gigawatts (GW) of energy storage worldwide were added, almost less than 30 % compared with 2018. The result is justified by the early maturity stage of some energy storage technologies – with presence in a few specific markets – and high dependence on support from appropriate policies ^1^. In the electrical sector, the energy storage can be applied to different goals: meeting the demand and reliability in the grid peak hours or as an asset on the liberalised electricity markets, benefit from price arbitrage depending on the fluctuation in spot prices, from capacity credit as transmission congestion relief or resource suitability, or for ancillary resources as voltage or frequency regulation and spinning or non-spinning reserves ^2^. On a smaller scale, these storage technologies can also directly benefit the consumer, namely, for solar photovoltaic (PV) self-consumption maximization, in the electricity shift from low demand to peak times or helping stabilize intermittent renewable energy sources (RES). They can also help in the demand-side management (DSM) or flexible power demand and in the smart-charging of electric vehicles (EVs) ^3^. Energy storage comprises different technologies, and in this work, the electrical sector application of a lithium-ion electrochemical energy storage technology will be discussed.

Lithium-ion battery (LIB) continues to be the most deployed electrical energy storage (EES) technology, driven mainly by the downward trend in costs ^4, 5^. Characterized by a) high efficiencies, b) moderated lifetime, c) low volume and weight per kWh of storage, d) temperature sensibility, and by being associated with low maintenance compared to other battery technologies, LIB are the state-of-art technology for electric vehicles (EVs) with across-the-globe investments by large market player battery manufacturers ^5^. It is a mature technology in the mobile device market, currently deployed in the automotive sector and is at an early stage in stationary applications. Nowadays, the automotive market is ten times greater than the grid-scale market. The research and development (R&D) efforts made and the diminishing costs of the EV batteries could boost the commercial and residential market. The search for alternative battery chemistries (post-lithium) to allow for better performance (power/energy rates, for example) could also offer a solution for the market, with possible declining battery costs. The ongoing scale-up allowed LIBs to present a downward trend regarding costs in the past years ^1, 4^, and they are forecasted to reach a cost potential of 70$/kWh in 2050 ^5^. The optimized cost reduction path goes along the further improvements of energy and economic indicators, such as the energy density, along the industrialization value chain.

A battery model is essential for designing and optimizing an electric power system and smart management. As non-ideal equipment, the battery response is affected by the state of charge or charge/discharge rates. The models allow for a more accurate analysis of the real system, which enriches the development of systems' analysis of performance and costs, allowing the faster application of commercial batteries in smarter residential markets. Predicting its performance allows estimating its use and durability, optimizing its energy use, and determining the applications that best fit the performance. Each microgrid equipment can be modelled, and the smaller the modelling error compared to the experimental performance, the better the description of the system's performance in real operating conditions. The traditional lithium-ion modelling approach generally relies on the existing models in MATLAB/Simulink. However, it is possible to identify some limitations of this modelling approach: the challenge in obtaining more accurate results (model is embedded in the code), difficulty in applying to another programming language, and verifying if the model aligns with the experimental result of the general battery. This work contributes to constructing a database with pre-defined experimental setups and validated models that allow the easier optimization of the use of a commercial lithium-ion battery, accessible to any residential user.

This work is focused on the LIB characterization testing and modelling for real-time application in future battery control scenarios and energy management strategies. In the literature, the modelling of LIB for EV application is broad, but for stationary applications, e.g., solar photovoltaic systems (validated with experimental results), the scientific literature found is scarce. This work aims to use a model to represent the dynamic behaviour of the battery with an adequate error, considering the interface with the power electronics and validating it through results experimentally obtained to allow the possibility of model integration into a more complex control system or algorithm. In the medium term, the developed model will later be integrated with models of other storage technologies to optimize the control and global operation of a complex but flexible and intelligent grid system, allowing it to respond to the objectives of future electrical networks.

This paper is organized as follows: Section 2 delivers a bibliographic literature review on lithium-ion battery technology and the existing modelling approaches that better describe the LIB in focus in this work. Section 3 presents the methodology used: the experimental microgrid description, the LIB and inverter characterization testing plan, and the model’s detailed description. Section 4 presents the results obtained, including the experimental data and the battery modelling results. Based on the methods used and the results obtained, a discussion is carried out in Section 5, followed by the conclusions of the work in Section 6.

## 2. Literature review

This section briefly describes the lithium-ion technology and the technology modelling approaches used throughout the bibliographic references.

### 2.1 The lithium-ion technology

The lithium-ion battery (LIB) was conceived and developed by the Japanese Asahi Kasei Corporation and released commercially in 1991 by Sony Corporation, followed by A&T Battery Co. in 1992 ^6^, especially for low-power portable applications. The technology was well accepted given its characteristics of high energy density, good performance, less heat generation, small dimension, lightweight (Wh/kg) ^7^, and no memory effect, compared with nickel-cadmium or nickel-hydride batteries. The low atomic number of lithium is the cause of the high electrode potential, which results in higher energy density. Over 90% of the worldwide production of LIBs is based in Japan, Korea and China ^8^.

Developing new high-energy-density lithium batteries has been challenging, requiring new anodes, cathodes, and nonaqueous electrolytes ^6^. Generally, a lithium-ion battery has two electrodes and an organic electrolyte, nonaqueous, containing dissolved lithium salts. The cathode is lithium metal oxide and the anode is from graphitic carbon. Inside the cell, the materials are ionically and not electrically connected by an electrolyte, and it has an insulating membrane. The reaction occurs with a characteristic electrochemical potential difference (voltage). LIB cathode materials can be associated with a variety of multiple chemistries, such as lithium cobalt oxide (LCO), lithium nickel manganese cobalt oxide (NMC), lithium manganese oxide (LMO), nickel cobalt aluminium oxide (NCA), and lithium iron phosphate (LFP). Recently, besides graphite, the anode can be composed of lithium titanate (LTO). NMC is the typical chemistry used in grid-scale ESS ^9^. Overall characteristics depend on the cell chemistry. However, it generally has higher gravimetric and volumetric energy density, high efficiency, high power capability, long cycle, and long calendar lifetime than other battery technologies ^10^.

LIBs allow fast and slow charging-discharging operation states and have high energy densities and good power densities. The technology has a battery management system (BMS) which monitors its general operating conditions – a range previously defined by the manufacturer) due to sensibility to high-temperature operation and high depth of discharge (DOD). Those factors are usually linked to faster degradation conditions (ageing), permanent damage, or unsafe operation. The response time of this technology is usually in the millisecond’s timescale, a fast response compared to the average of other battery technology types. It is also easily scalable in terms of power or energy. In recent years, R&D has evolved using non-flammable and /or flame-retardant additives (non-flammable electrolytes) ^6^. LIBs are sensitive to temperature. Usually, an active cooling system is integrated within the building/container of the battery (or in the EV refrigeration battery system) to reach its optimal temperature range (or move away from extreme temperature ranges). Generally, LIBs are designed to operate at about 21°C so that a heating-cooling active system can be used.

Improvements of this technology are related to its core aspects as energy storage, mainly from its competitiveness in the market since they are still associated with a high production cost. The technology still presents challenges in the 2 ^nd^ life usage or at the end-of-life /recycling process, being its salvage value lower than the processing cost ^11^. After high-intensity applications, as in EVs application, LIBs present generally good condition to be further used in high energy density applications, such as in grid storage ^12, 13^. LIB recycling is limited at present, having recycling figures below 3% ^14, 15^, but this will be a vital issue in its future deployment. Different LIB technologies are recycled through similar processes to recover materials like lithium, copper, cobalt, nickel, iron, aluminium, and manganese. The level of toxicity of the substances used in LIB is lower than other battery technologies, and in some countries, these are still disposed of in landfills ^15^. For LIBs, lithium appears to be the only critical raw material. In contrast, other critical elements are being studied to reduce their need to incorporate the battery, e.g., the use of manganese instead of critical cobalt is expected to be used for electrode coatings in the future ^16^.

***2.1.1 Standards for battery operation***

The following standards are the currently most relevant in force for the technology:

▪ UL 1642 ^17^ is a standard that expresses guidelines for manufacturers, with procedures on construction, performance, and electrical, mechanical, environmental, and marking tests.
▪ LIB’s transport guidelines are described in IEC 62281 and ST/SG/AC.10/27/ ^18^, with the United Nations recommendations.

Furthermore, the general standards for secondary batteries are highlighted in the following:

▪ UL 2054 ^19^ is a reference for household and commercial batteries to understand the considerations made for residential sector application, regarding construction and testing for the electrical, mechanical, enclosure, fire, and environmental performance and conditions, and marking.
▪ IEC 61427-1 and IEC 61427-2 refer to the photovoltaic off-grid and on-grid applications, respectively, and IEC 62933-5-2 describes the safety requirements for grid integrated EES systems – electrochemical based systems ^18^.

Policies and the regulatory framework for batteries are still under development. For instance, the European Parliament is currently preparing the proposal for a regulation on batteries and waste batteries, including raw materials, carbon footprint and end-of-life handling, setting the sustainability requirements for EES technologies ^20^. The regulation is missing for different application scenarios. Critical aspects must be addressed regarding aspects of the integration, testing and operation of this technology, such as the controls and BMSs, temperature, degradation and safety measures.

### 2.2 Modelling a lithium-ion battery

LIB models are being emphasized in the current battery’s panorama ^21– 25^, most of them fostered by the research in the automotive sector field ^26– 28^. A battery model predicts the performance of the battery unit to be used on a simulation framework, allowing the optimization of the system itself and its integrated control within a microgrid. Generally, it starts at a single-cell level, progressing to a unit system description, using the nominal energy capacity and voltage characteristics. The efficiency losses due to racking, hardware for control and safety, and power converter elements should also be considered. The voltage curve depends on the battery state of charge, operating current, resistance, and energy capacity. The internal resistance increases over time, conducting to a decreasing usable voltage range. Operating the battery at higher currents is related to a higher rate of voltage decrease, reducing the available bulk capacity. The thermal behaviour could be described using a heat transfer coefficient with the environment for instantaneous thermal effects on capacity and resistance. The temperature effect is usually described as a function of ambient temperature and operating current, resulting in a resistance parameter.

A brief review of available LIB models was made, and different approaches were found. In article ^29^, a description of LIB models and parameter identification techniques is made; the authors of 22 compared different LIB models; in reference 28 the authors present LIB models for automotive applications, and the authors of 21 provide a review of models for generic batteries. Modelling approaches based on MATLAB/Simulink are presented in studies ^27, 30, 31^. Battery testing based on pulse-charging is carried out by the authors of 32 and by the authors of 33. In the case of model approaches emphasising on chemical modelling, the authors of 34 explored parameters identification techniques for a LIB model. In 24, an extended Kalman filter is used to estimate the SOC of a LIB. The authors of 35 estimate parameters of the electric model, such as the resistances and capacitors, directly from measurements.

LIB models research in the literature outnumbers those for stationary applications, justified by the EV's continuously increasing deployment. The design of future smart grids relies on robust battery modelling and field validated approaches. Many research activities are devoted to optimising energy efficiency and energy management for smarter energy systems and designing scenarios for battery applications, such as in buildings or EV charging stations, where the battery performance influences the investment. The generated model can be replicated and, among the possibilities, be used to study different energy management strategies, such as PV ramp-rate relief or energy arbitrage or investigate the possible integration with other energy storage technologies within microgrid or building scenarios.

The voltage equation modelling is based on the charging and discharging efficiency. Three main approaches are found in the literature: Shepherd’s battery model, electric circuit models, and the modified Shepherd’s battery model. These three approaches are briefly presented below.

**Figure 1.**
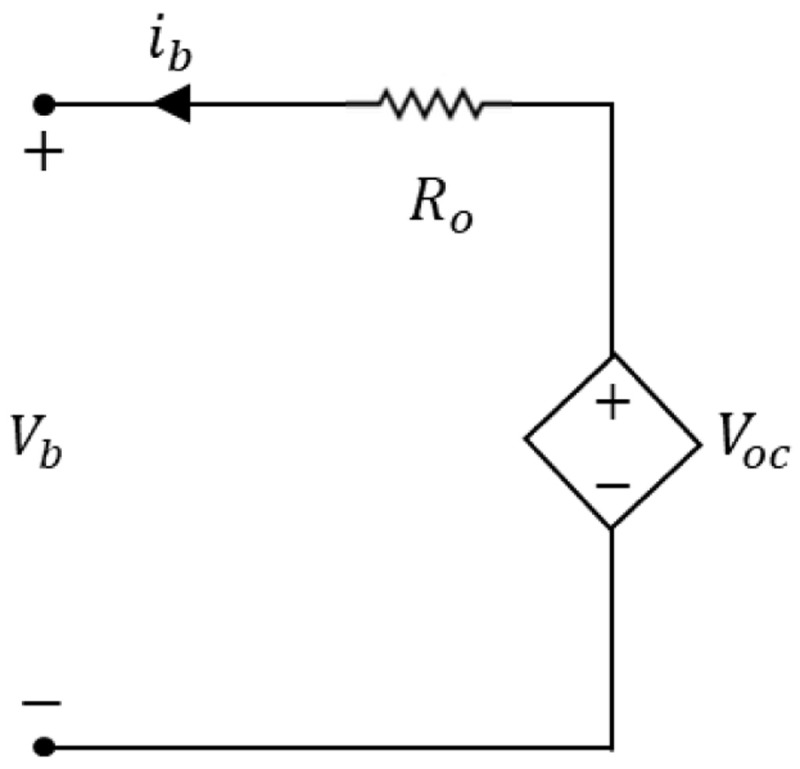
Equivalent circuit representation to the lithium-ion battery model.

***2.2.1. Electric circuit models (ECMs).*** Equivalent electric circuit models can be applied to describe different battery technologies ^21^. The ECMs for lithium-ion batteries found in the literature review showed satisfactory output results, following simple and fast algorithms and representing the battery in permanent and transient states ^27, 31, 35^. Their suitability for stationary applications is considered a good approach. The model shown in Figure 1 has a constant voltage source in series with a resistor and is the straightforward ECM representation.

The current *i _b_* represents the dynamic internal current, *R _o_* represents the internal ohmic resistance, *V _b_* is the battery voltage in its terminals, and *V _oc_* is the applied input voltage ^21^. The battery voltage is obtained through the simple circuit analysis of this equivalent circuit, expressed in Equation (1).


$$\;V_{battery}(t)=V_{oc}-R_{o}i_{b}(t)\;(1)$$

Among the ECMs, it is possible to find simple models, Thevenin-based models, impedance-based models, combined ECM and generic based models ^21^. The presented model includes the determination of the SOC as a function of the open-circuit voltage (V _OC_), and others could also include bias effects. Although more accurate, capacitors represent significant additional computational times.

***2.2.2. Shepherd’s battery model.*** Shepherd’s battery model is a widely known model that describes a battery’s terminal voltage over the current inputs. It is generally described through a constant current discharge, expressed in Equation (2) ^27^,


$$\;V_{b}(t)=E_{0}-K\frac{Q}{Q-it}i(t)-R_{o}i(t)\;(2)$$

Where,

*V _b_* is the terminal voltage of the battery, in V, at instant *t*.

*E*_0_ is the battery constant voltage, in V.

   K is the polarization constant, in *V*/ *Ah*.

   Q is the battery energy capacity, described in units of Ah.

*it* is the discharge energy capacity, in Ah.

*R*_0_ is the battery internal resistance, in Ω.

*i*( *t*) is the dynamic current (A) in instant *t*.

**Figure 2.**
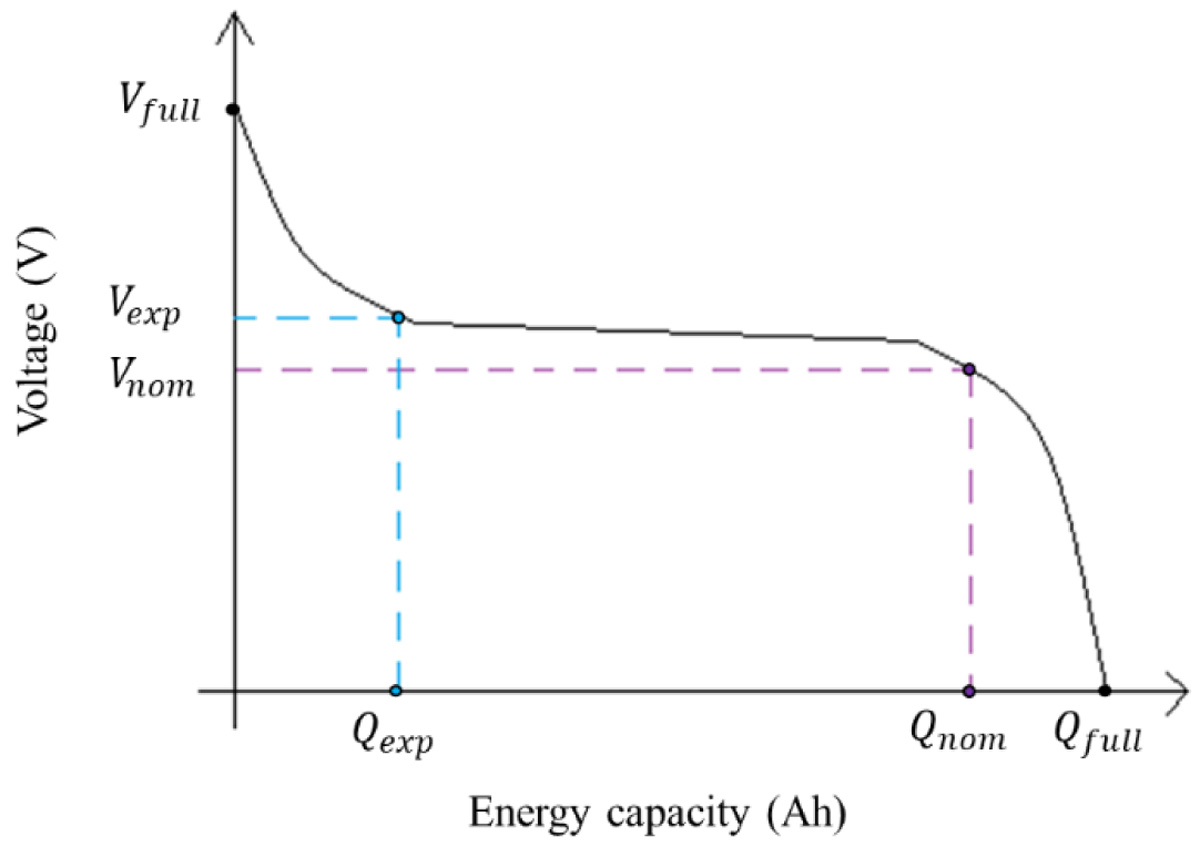
Example of an ideal discharge curve, where the three points can be extracted: the ( *V _full_*, *Q _full_*) are the voltage (V) and energy capacity (Ah) of the completely charged battery; the ( *V _exp_*, *Q _exp_*) are the voltage (V) and energy capacity (Ah) at the end of the exponential zone; and ( *V _nom_*, *Q _nom_*) are the voltage (V) and energy capacity (Ah) at the end of the nominal zone. This image has been reproduced with permission from 27.

The voltage equation parameters – *E*_0_, *K*, *R*_0_ – can be obtained through the relation established on three points of the battery discharge curve given by the manufacturer (( *V _full_*, *Q _full_*), ( *V _exp_*, *Q _exp_*) and ( *V _nom_*, *Q _nom_*)), illustrated in Figure 2, below shown.

Shepherd’s model is usually studied in the literature to describe the automotive battery behaviour.

***2.2.3. Shepherd model modified versions.*** Shepherd’s battery model can be modified to better describe the dynamic battery behaviour through an exponential function, previously presented in the work developed by the authors of ^31^. The modified Shepherd’s model charge and discharge voltage curves are based on Equation (2) and are represented below in Equation (3) and Equation (4).


$$V_{batt\_ch}(t)=E_{0}-K\frac{Q}{it-0.1Q}(it+i(t))+Ae^{-B\;it}-R_{o}i(t)\;(3)$$

$$V_{batt\_dis}(t)=E_{0}-K\frac{Q}{Q-it}(it+i(t))+Ae^{-B\;it}-R_{o}i(t)\;(4)$$

Where, *V _batt_ch_* represents the charge voltage and, *V _batt_dis_*) represents the discharge voltage. The rest of the parameters present in Equation (3) and Equation (4) ( *E*_0_ K, A, B) can be determined through the manufacturer discharge curve and are directly determined with the use of the equations in Table 1, shown below.

**Table 1.** Parameter’s description of equation 3 and equation 4.

Model parameter	Description	Equation representation
A, Exponential Voltage Amplitude Constant, in V	The amplitude of the exponential region.	*A* = *V*_*full*_ – *V*_*exp*_
B, Time Constant Inverse, in *Ah* ^–1^	Charge at the end of the exponential zone of the battery’s discharge curve. The scalar value of 2.3 was used in 31 to improve the fit to the specific battery used.	$B=\frac{2.3}{Q_{exp}}$
K, Polarization Constant, in *V/Ah*	Calculated using *V*_*full*_ and the end of the nominal zone of the discharge curve. The scalar value of 0.065 was used in 31.	*K* = *X*[ *V*_*full*_ – *V*_*nom*_ + *A*( *e* ^*–BQ*^_*nom*_) –1)] $\frac{Q_{full}-Q_{nom}}{Q_{nom}}$
R, Internal Resistance, in Ω	The internal resistance of the battery at steady-state current. *υ* is the efficiency of the battery, and *V*_*nom*_ and *Q*_*nom*_ are the nominal values for voltage (V) and energy capacity (Ah), respectively, of the curve (see Figure 2).	$R=V_{nom}\left(\frac{1-\upsilon }{0.2\times Q_{nom}}\right)$
*E*_0_, Battery Constant Voltage, in V	Represents the value when the battery is close to completely discharged and no current is flowing.	*E*_0_ = *V*_*full*_ + *K* + *R* × *i* – *A*

The three points of the discharge curve are used (see Figure 2) to calculate the voltage-current curve. In the work developed by the authors of 31, the proposed model considers this approach, including the battery lifetime. Moreover, the authors test the model within a SOC operating range in the linear region of the battery discharge curve (to operate within the 20–85 % SOC range) and consider the temperature operating range to be maintained within the -10°C to 45°C range.

## 3. Methods

**Figure 3.**
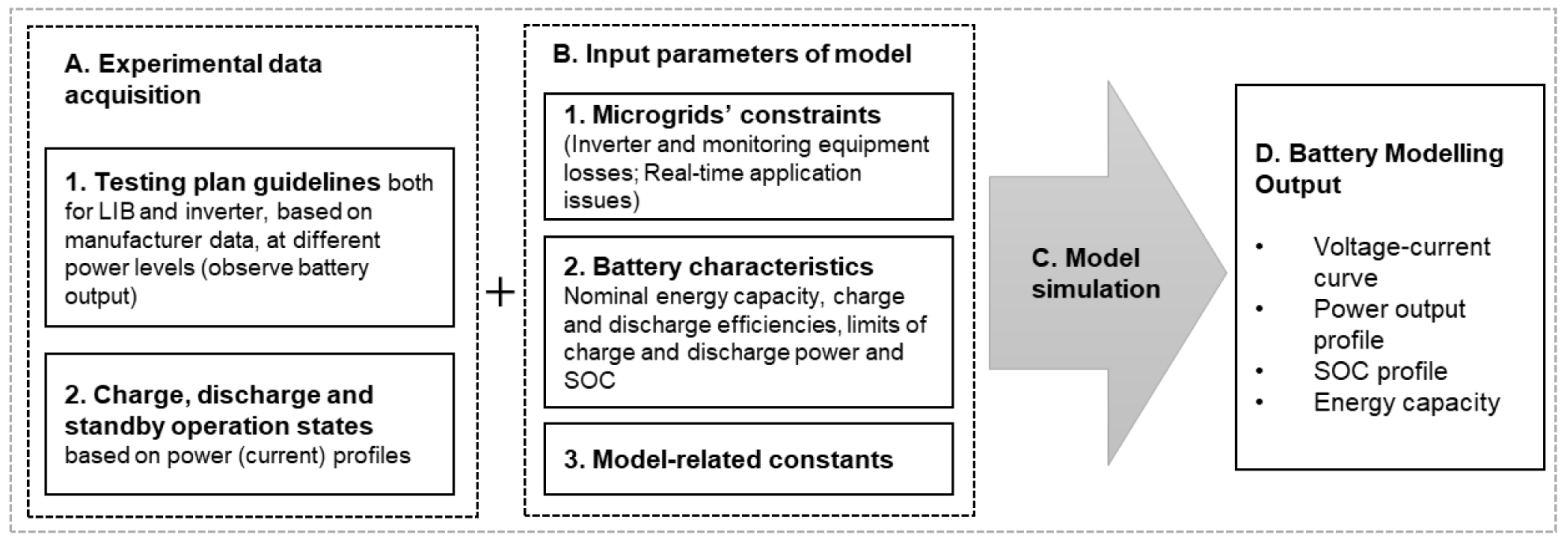
Methodology to describe the lithium-ion battery under study. LIB, lithium-ion battery; SOC, state of charge.

 In this work, the authors aim to develop a model for a commercial LIB that allows its real-time description for stationary applications. An experimental setup is developed to characterize the battery, and further model validation against the experimental data acquired is carried out. The methodology follows the steps described in Figure 3, which details the input parameters subjacent to the model construction and development.

The following subsections describe the key aspects of the experimental data acquisition and the model approach.

### 3.1. University of Évora microgrid infrastructure

**Figure 4.**
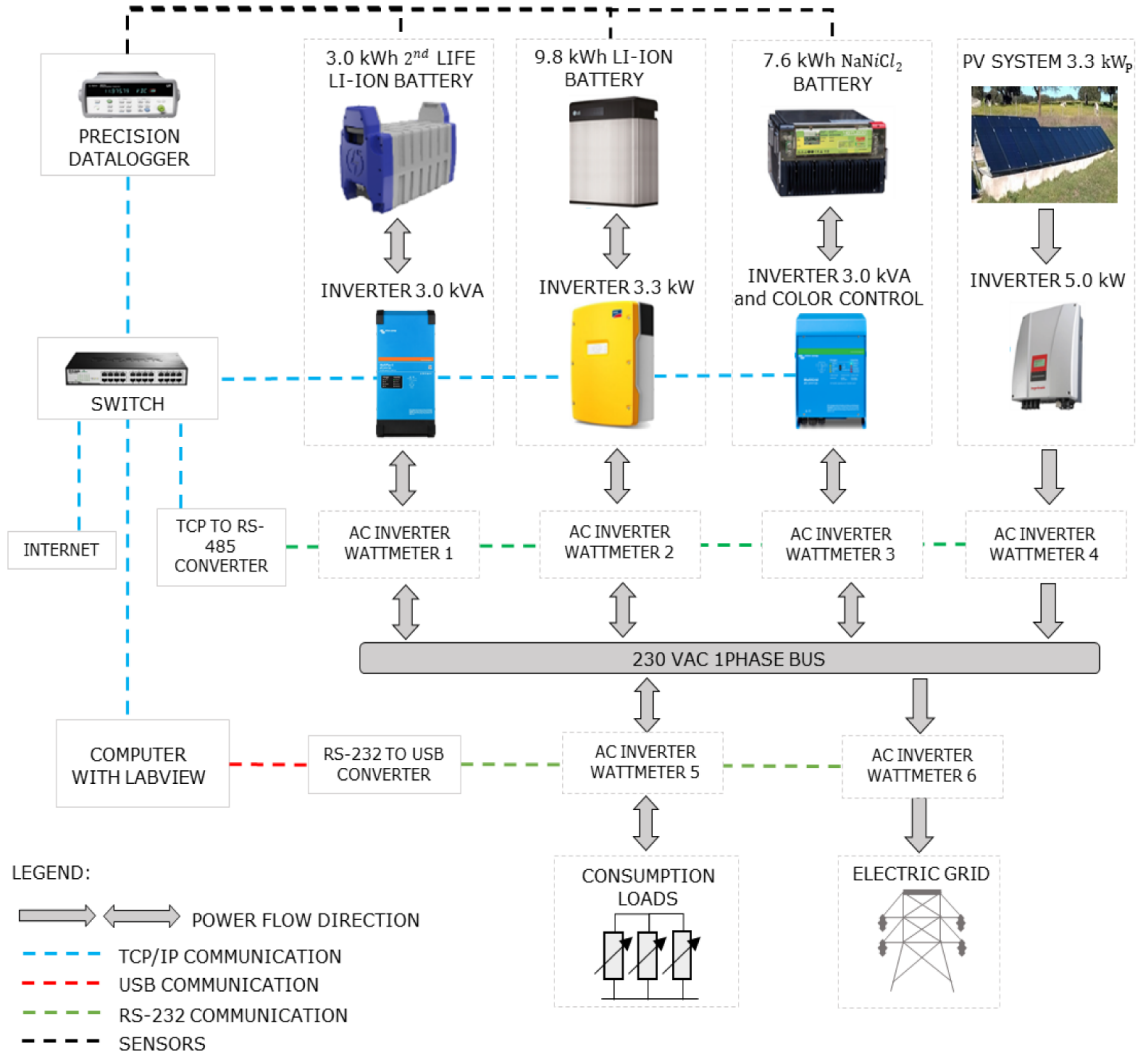
General schematic of the equipment and control platform of the microgrid of the Renewable Energies Chair at the University of Évora. In the figure, PV is the solar photovoltaic generation; AC is alternating current; TCP/IP is the Transmission Control Protocol/Internet Protocol (to allow communication on a network); RS-232 is a protocol for the exchange of data (generally used in the serial ports of the computers).

The LIB characterization process aims at verifying the performance and dynamic behaviour of the selected battery: 5.0 kW/ 9.8 kWh (189 Ah) LIB energy storage from the LG Chem manufacturer ^36^, model RESU 10, with a nominal voltage of 48 V. The LIB and the SMA Sunny Island ^37^ 4.4M inverter (3.3 kW nominal power) are integrated into one of the University of Évora (UÉvora) microgrids. The integration consisted of connecting the systems to the grid, installing AC meters, DC measurement devices and temperature sensors (in the battery’s two surface parts – above and lateral - and environmental temperature). The next step consisted in establishing a communication set with the inverter through the Modbus TCP/IP protocol (see Section 3.2 for further details). The current microgrid schematic is shown in Figure 4, including a 3.3kW solar amorphous photovoltaic installation, a 3.0 kW/ 7.6 kWh sodium-nickel chloride battery, and a 3.0 kW/ 3.0 kWh 2 ^nd^ life lithium-ion battery, and monitoring equipment (AC and DC meters and temperature sensors) on each relevant point of the microgrid. This research infrastructure was designed for the systems’ operation study and development, where a communication and control platform were developed. The manufacturer data ^38^ of the commercial battery is displayed in Table 2.

**Table 2.** Reference operating conditions and main characteristics available by the manufacturer of the LG Chem Resu10 48 V LIB ^38^.

Variable	Unit	Value
General		
DC voltage operating range	V	42.0~58.8
Volume (exterior box)	*m* ^3^	0.05
Weight	Kg	75
Average environmental temperature	°C	20
Depth of discharge	%	80
Energetic		
Nominal energy / useful	kWh	9.8 (25°C, 100% SOC) / 8.8
Nominal power / maximum / peak (in 3 s)	kW	3.3 / 5.0 / 7.0
Nominal Capacity	Ah	189
Maximum Current	A	119 at 42V
DC nominal Voltage	V	51.8
Cycles number (90% DOD, 25°C)	-	6000
Cycles number (80% DOD, 25°C)	-	10000
Environmental conditions		
Cooling	-	Natural convection
Operating Temperature	°C	-10 a 45
Optimal Operating Temperature	°C	15 a 30
Storage temperature	°C	-30 a 60
Humidity	%	5 a 95 (no condensation)
Altitude	m	< 2000

### 3.2. LIB and inverter testing description

In the consulted bibliography, battery testing procedures generally consist of a battery discharge at a constant current, known as C-rate ^27, 31^. It is generally encountered in literature for the batteries' description to normalize it with the battery capacity, which might be variable. The test consists of discharging the battery at a calibrated current relative to its maximum capacity, which led the battery modelling to be developed based on the manufacturer discharge curve at constant current, one of the most widespread models found in the literature ^27, 31, 39^. In the absence of both a manufacturer curve and the option of testing the LIB at a fully controlled constant current discharge (due to the inability to control all the inverter parameters, which satisfies the relation of power-voltage-current at each instant), the charge and discharge curves were obtained through the available points of control of the battery inverter, which is the alternating current (AC) delivery point. This case consists of the battery testing procedure with an E-rate ^40^ - measuring the power rate at which a battery is discharged relative to its maximum capacity, in Wh.

The experimental tests and respective data acquisition are presented in the following paragraphs. The outlined test plan considers the manufacturer's operating conditions (see Table 2), with a 20–90% SOC range. The operation of the battery within the referred range includes a safety margin closely related to the depth of discharge (DOD) and the technology degradation. The air-conditioning unit controls the environment temperature within 15–25°C.

**Figure 5.**
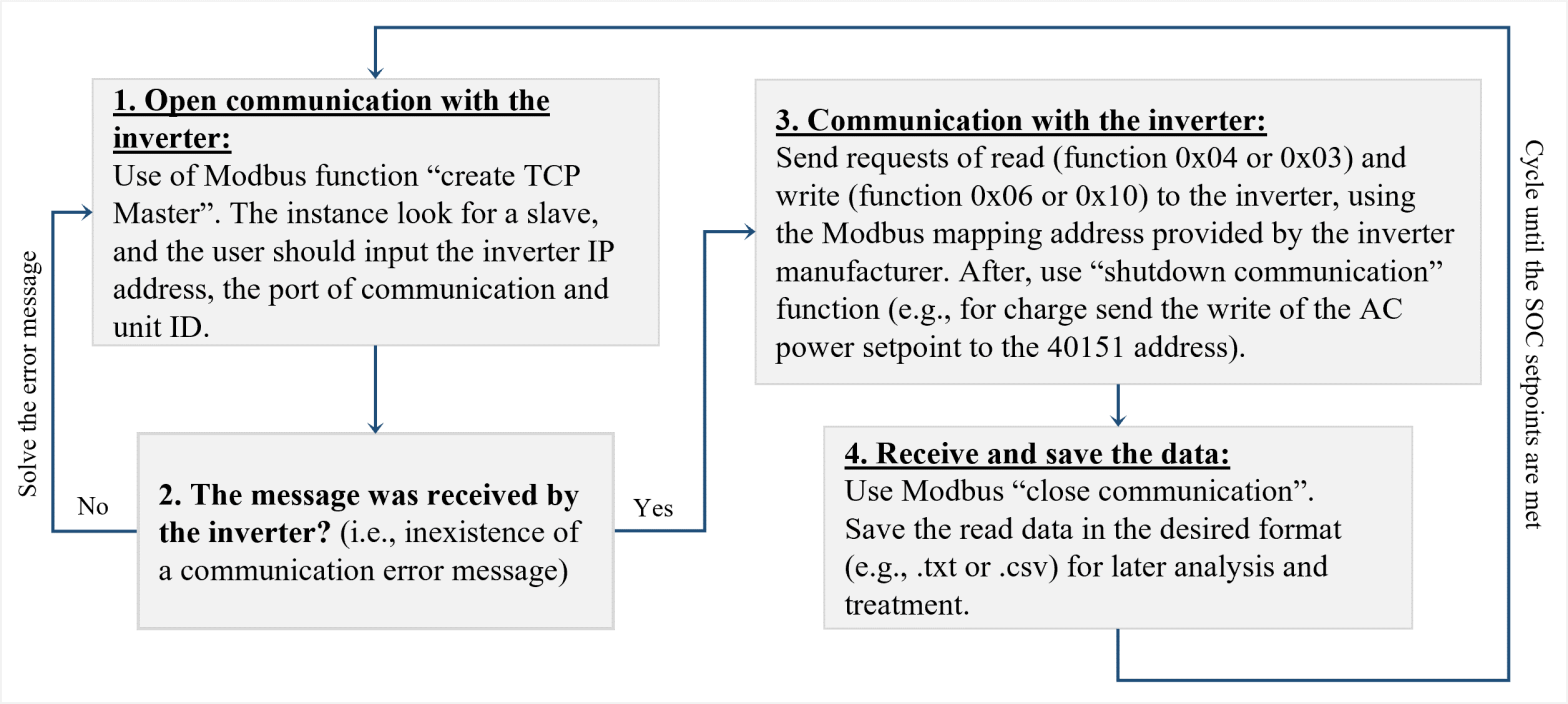
Communication establishment with the inverter to perform the charge-discharge tests.

A battery-inverter communication control was developed to conduct the testing plan and real-time data monitoring. The communication is based on the Modbus TCP/IP protocol ^41, 42^, with the help of the LabVIEW 2014 programming environment (the communication can be established in a similar routine through open-sourced software such as Python). In that sense, the inverter needs to be connected to the Ethernet, detaining an IP address. It relies on the Modbus mapping addresses available from the inverter manufacturer, SMA Sunny Island 4.4M ^42^, that is available from reference ^42^ (“Technical Information - Modbus® Interface”, and consult the .xlsx file choosing the model of the inverter). Through the communication establishment, the inverter is asked to, periodically (at 2-second intervals), retrieve and send commands (based on the power profiles at the AC point connection). An example of the communication establishment is detailed in Figure 5.

The data acquisition is achieved by reading the parameters of the inverter from the high precision AC wattmeter ^43^, and the measuring precision datalogger ^44^. The acquired parameters include current, voltage, power, temperatures, and battery and inverter alarms.

**Figure 6.**
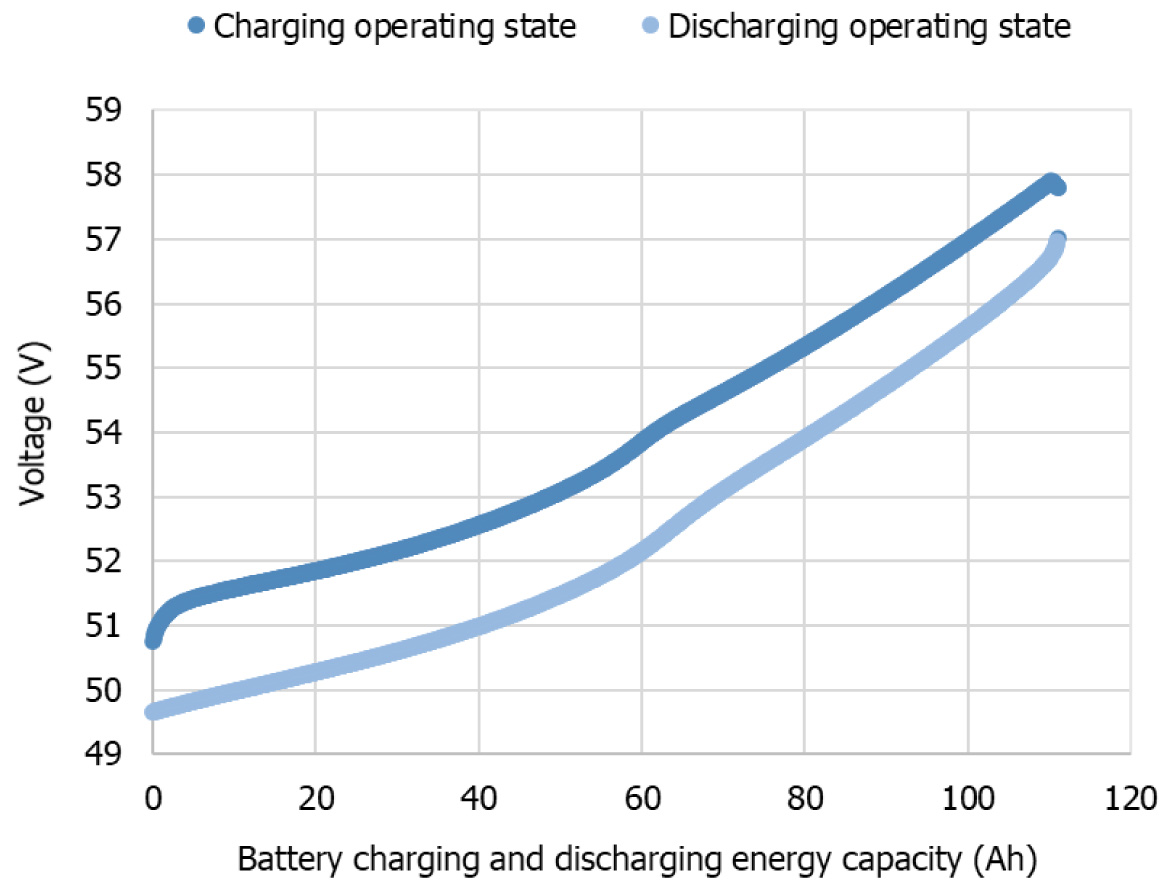
Complete battery charge-discharge cycle at a constant power level of 2.7 kW at the alternating current delivery point, from 20–90 % state of charge range.

The battery cycling tests were conducted after assuring reliable data measurement acquisition and optimization of the control program (definition of timeframe, prioritization of specific readings, organizing the different equipment readings). The cycling corresponded to about 30 complete charge-discharge cycles at predefined constant power levels throughout the SOC range (from nominal power to low operating power: 330-3300). The obtained average data for each power level is made available in *Underlying data* ^45^. An example of a full charge and discharge battery test is shown in Figure 6, at a constant power level of 2.7 kW, throughout the mentioned SOC range.

The inverters operate at different power levels and according to a power-related efficiency profile. Given the integrated battery-inverter use, it is essential to consider the efficiency curve of the inverter, which varies throughout its power range (either charging or discharging). For that reason, it is relevant to acquire its experimental efficiency curves since they will impact the overall battery system results.

To evaluate the performance of the SMA Sunny Island 4.4M inverter, a test control was implemented in the LabVIEW 2014 programming interface, fully reproducible through open-source software, e.g., Python. It used Modbus TCP/IP and was achieved by the mapping of addresses given by the inverter manufacturer (in this case, SMA) ^42^. The test control made was similar to the previous, where only the remaining time at a certain level of power was variable. The protocol consisted of operating the inverter at different rates of its nominal power (5% to 100% rate) during periods of 15 minutes. DC and AC electrical parameters were monitored. The test’s main objective was to calculate the charge and discharge efficiencies over the power operating levels, presented in the next section of this work ( Section 4).

### 3.3. LIB modelling application

Using the previously obtained experimental data to describe the LIB, a LIB model was developed considering modelling adaptations of Shepherd’s model, described in Section 2.2.3. The authors of 31 describe the battery through the manufacturer-provided curves. In the present work, the battery is described by the results of the experimental testing at different constant power levels over the complete charging and discharging operating states. The modified version of Shepherd's model was computed to obtain the dynamic battery behaviour, using Equation (3) and Equation (4), considering the dynamic current over the state of charge range at a particular power level.

In addition, the state of charge parameter of the battery at each instant t is generally estimated by the following Equation (5) and Equation (6),


$$SOC(t)=SOC(t-1)+\frac{i(t)\times \text{Δ}t}{Q}\;,\;\text{if}\;\text{Q}\;\text{is}\;\text{in}\;\text{the}\;\text{unit}\;\text{of}\;\text{Ah}\;(5)$$

$$SOC(t)=SOC(t-1)+\frac{P(t)\times \text{Δ}t}{Q}\;,\;\text{if}\;\text{Q}\;\text{is}\;\text{in}\;\text{the}\;\text{unit}\;\text{of}\;\text{Wh}\;(6)$$

Where Q is the total energy capacity of the battery, and the Δ *t* is the difference of the step time: *t* – 1 (last instant of *t*) and *t*. The *Q* values are bounded by its real defined range of minimum and maximum energy capacity values. Using the model described in Section 2.2.3 to describe the LIB understudy better, some adaptations were made. In Equation (3), a multiplication factor of 0.65 for Q was used instead of the 0.1 described. Regarding the rest of the parameters, the used time constant inverse of 2.3/ *Q _exp_ Ah* ^–1^ was maintained, and a polarization constant with a scalar value of 7.3 was used instead of 0.065 (see Table 1). For the obtained voltage to be described by Equation (3) and Equation (4), the curve has a constant fit of a scalar value of +41.2 (obtained through the observation of a mismatch of the initially computed voltage curve and experimental voltage values obtained).

The model was computed using MATLAB (2017b) programming and is fully reproducible through alternative software, e.g. Python (see *Software availability* ^46^). As a first approach, the operation of the battery at constant power levels, acquired experimentally, was chosen to validate it. The model was used to suit both charge and discharge curves from the experimental values, given the need to describe the overall battery behaviour. For the intermediate power values, a technique of regression fittings was used, taking advantage of the MATLAB fitting tool (also existing in alternative software, such as the open-sourced software Python).

The proposed model uses the current as input to represent the LIB voltage behaviour. Regarding the practical model application within a more extensive model regarding all microgrid systems (and to implement energy management strategies), it is generally helpful to have the battery output in terms of the energy and power domains. The cp-rate can be defined by the rate of constant power that will cause the battery to discharge in a certain amount of time, as explained in 31.

***3.3.1. LIB ageing model.*** The battery lifetime depends on the operation conditions based on temperature, SOC and total energy throughput (electrochemical operating ranges), charge and discharge rates ^47^ and the total number of cycles. In the case of the present work, particular emphasis will be given to the description of the battery in its current state through the validation of the model approach against the experimental results. The main goal is to have the battery model in its current operational state, validating this model approach with the experimental results. Nevertheless, the LIB ageing effects should be included in the modelling used for real-time predictive optimization control modelling for wider timeframes of simulation. There is no standard model for LIB ageing, although this model should describe the fade mechanisms triggered by cycling patterns, storage, and others. Lifetime fade or degradation (capacity decrease) and cycling fade affect the performance and battery lifetime, impacting its financial results (given the high upfront cost).

**Figure 7.**
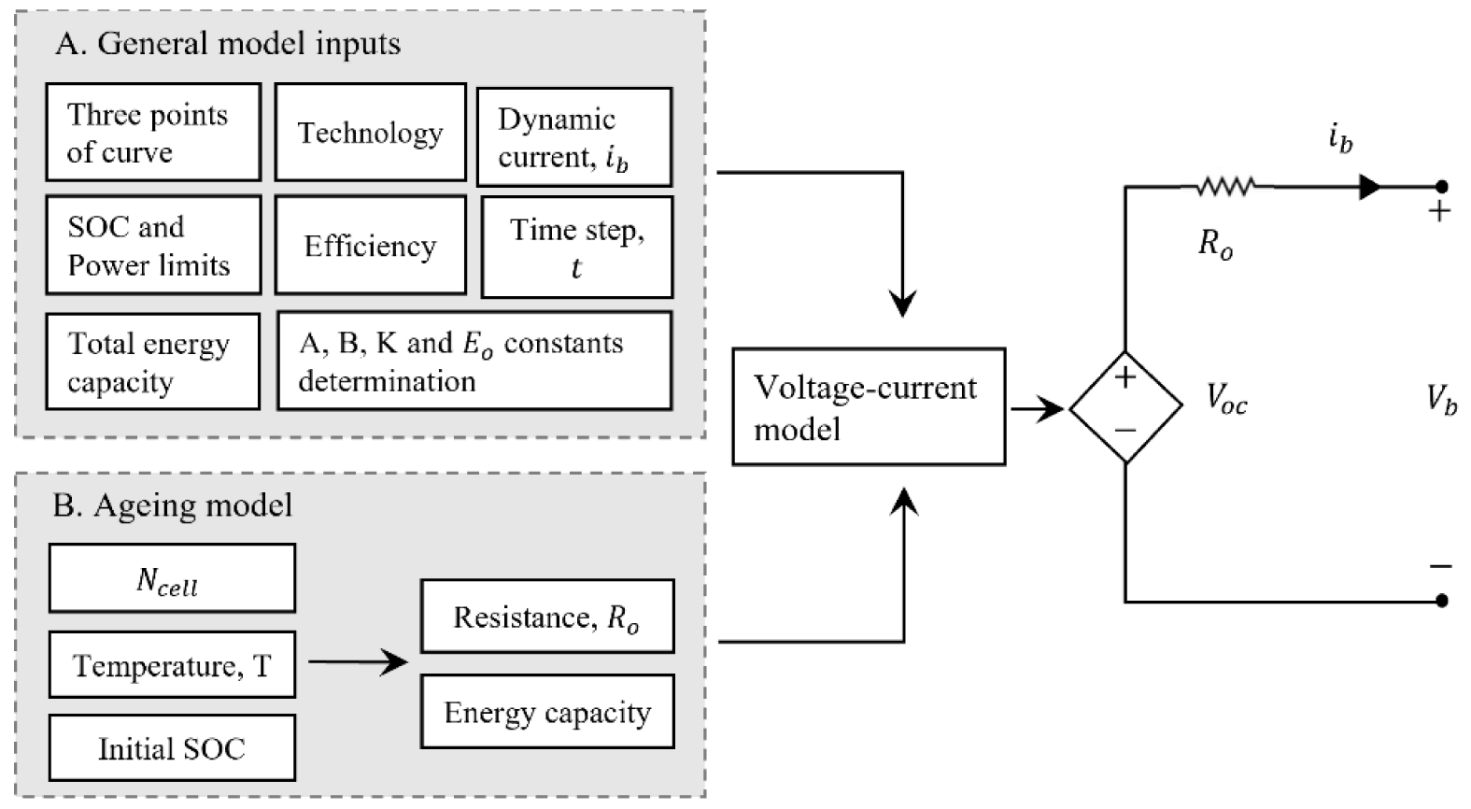
Complete model of the lithium-ion battery technology approach. In this figure, A is the exponential voltage amplitude constant (V); B is the time constant inverse (Ah); K is the polarization constant (V/Ah); *E*_0_ is the battery constant voltage (V); *N _cell_* the number of cells in series; SOC is the state of charge of the battery; *R*_0_ is the series resistance of the equivalent model (Ω); *V _oc_* is the open-circuit voltage (V) and *V _b_* is the battery terminals voltage (V).

The present work approach allows for updating the values of internal resistance (depending on temperature and state of charge) and energy capacity (depending on temperature, T, and cycle count, *N _cell_*) based on the National Renewable Energy Laboratory (NREL) lifetime model ^47^. This model will be included in detail in further work application of this model. The final complete LIB long-term use model is shown in Figure 7.

## 4. Results

This section summarizes the main output results of this work, firstly concerning the battery pack and inverter characterization performance, and secondly, the developed model which fits the experimental data previously obtained.

### 4.1. Battery testing and inverter results

Based on the methodology described in Section 3, the battery and inverter characterization data were obtained, and the battery performance indicators were calculated (see *Underlying data* ^45^). The characterization included the realization of at least three cycles for each power level to obtain greater precision and accuracy within the results.

**Figure 8.**
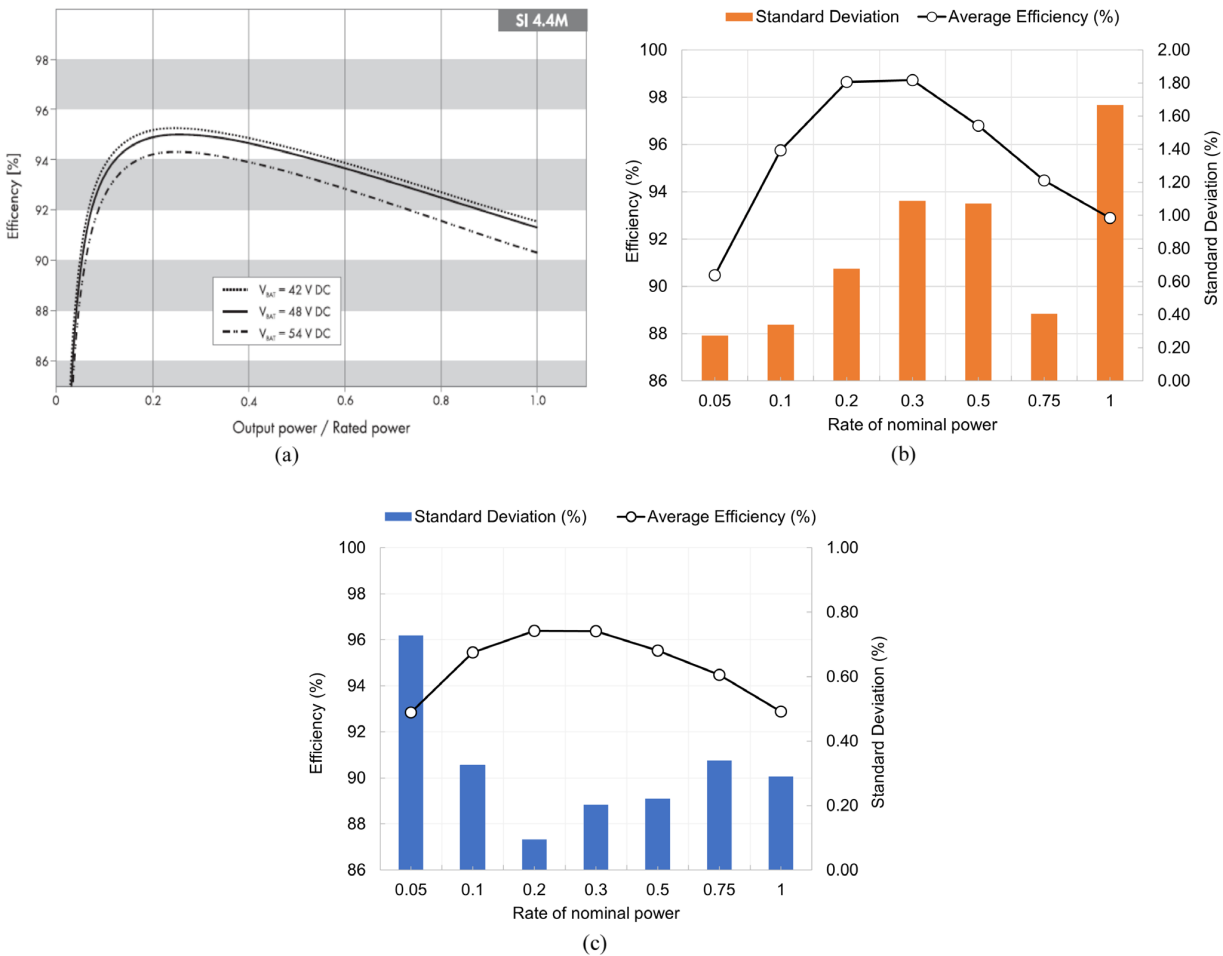
Inverter efficiency curve as a function of the power level ( **a**) given by the manufacturer ^48^, ( **b**) from the experimental results of discharge, and ( **c**) from the experimental results of charge. In this figure, ( **a**) has been reproduced with permission from 48.

Concerning the inverter charge efficiency (coulombic), it was possible to obtain an average value of 94.8 %, with an STD of 1.4, and regarding discharge, the obtained average value was 95.6 % with an STD of 2.7. The efficiencies defined by the manufacturer are an IEC efficiency of 94.0 % and a maximum efficiency of 95.3 %. Figure 8 presents the data from the inverter manufacturer in the technical document (a) ^48^ and the experimental data obtained for different power levels for discharge (b) and charge (c).

Regarding the battery, the charge and discharge efficiencies calculation was based on the DC measurements acquired throughout the tests, presented in Table 3.

**Table 3.** Charge and discharge battery cycling results, at constant values of power over the state of charge range.

Rate of nominal power	AC Power (W)	DC Energy	
		Average efficiency	Standard deviation
10	330	0.75	0.05
15	500	0.84	0.01
20	650	0.85	0.00
30	1000	0.84	0.07
50	1650	0.90	0.03
60	2000	0.95	0.10
75	2500	0.96	0.05
80	2700	0.79	0.00
100	3300	0.74	0.01

**Figure 9.**
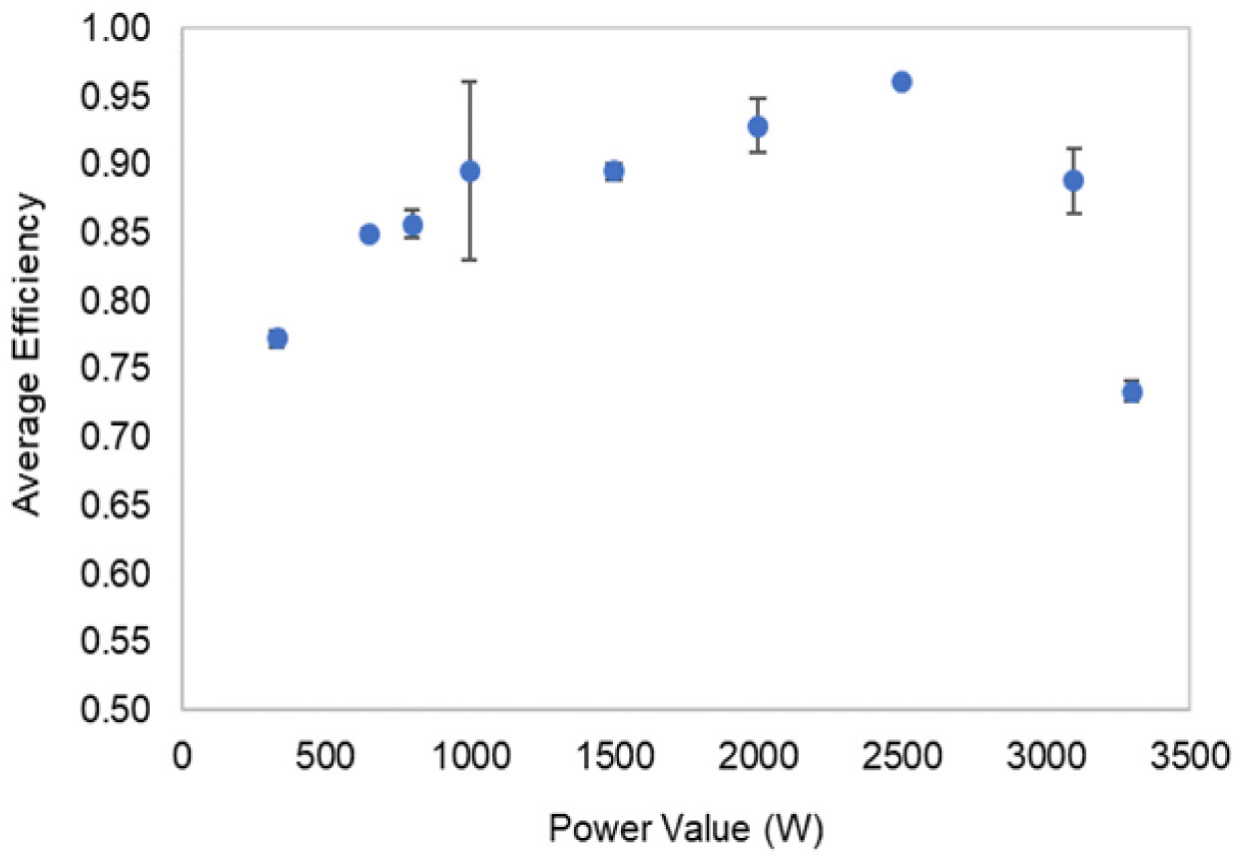
Battery efficiency at each level of power, and respective standard deviation results.

Figure 9 presents the average battery efficiency at each power level and the correspondent STD. The experimental results made it possible to obtain an overall average battery efficiency of 84.6 % with an STD of 7 %. In Figure 9, the influence of the inverter efficiency dependency on power rating is noticeable.

The tests performed allowed the calculation of other battery indicators besides efficiencies, such as energy capacities, energy densities, full charge and discharge spent times, response times, and typical discharge power. These indicators are presented in Table 4.

**Table 4.** Battery performance indicators resulting from the experimental tests.

Battery Performance Parameters	Units	Results
Total Capacity (Charge capacity)	kWh	6.6 ± 0.5
Useful maximum capacity (Discharge capacity)	kWh	5.5 ± 0.4
Energy Density in charge by unit of mass	Wh/kg	86 ± 6.0
Energy Density in discharge by unit of mass	Wh/kg	73 ± 5.0
Energy Density in charge by unit of volume	Wh/L	129 ± 10
Energy Density in discharge by unit of volume	Wh/L	109 ± 8.0
Fastest/slowest charge	h	2h04min / 10h46min
Fastest/slowest discharge	h	1h33min / 3h48min
Charge/discharge efficiency (complete cycles)	%	84.6 ± 0.1
Charge/discharge maximum power	kW	3.3
Response Time	Seconds	< 1s
Typical discharge time	h	Hours

**Figure 10.**
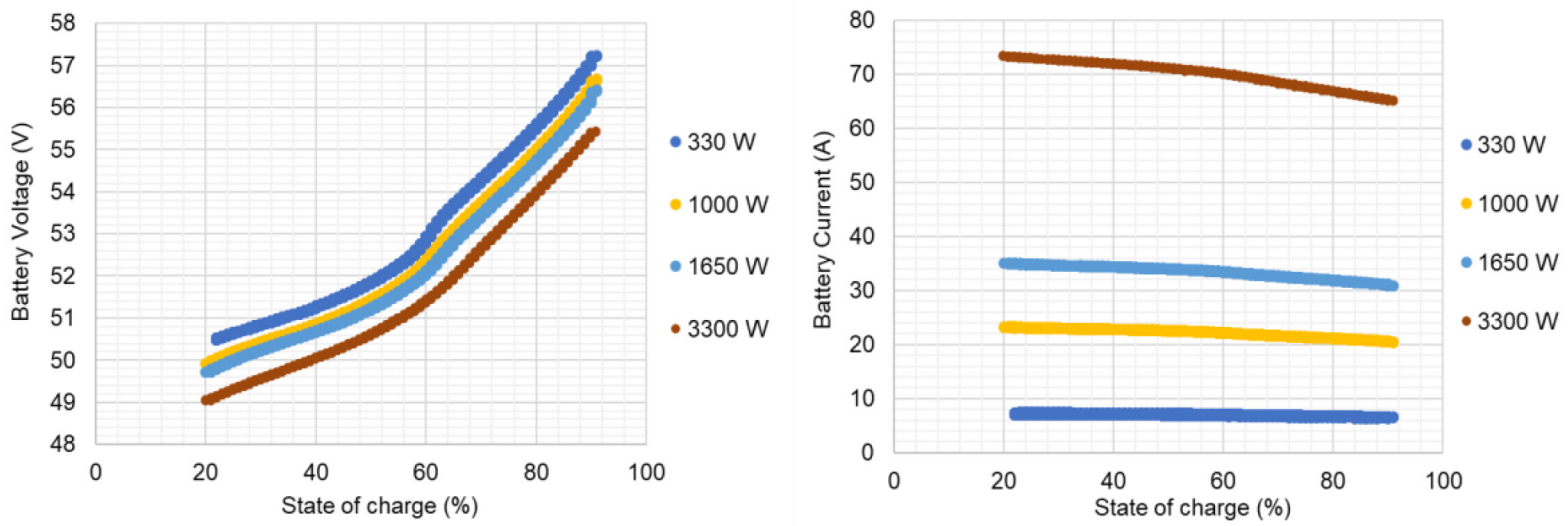
Lithium-ion battery voltage and current data from the experimental test plan, for complete charge-discharge cycles, at different constant power levels (due to readiness, only few power levels are represented).

The experimental acquired voltage-current data in function of the battery energy capacity (or SOC) were used to validate the modelling approach of the following Subsection (4.2). Figure 10 exemplifies some of the experimental results obtained.

### 4.2. Modelling approach to the overall battery operating states

In the literature is common to find references on the battery modelling for one C-rate ^27, 31^ or one E-rate ^40^. However, it lacks a solution to describe the battery behaviour over its overall operating frame required to meet the aim of this work: the use for smart power applications’ management. The validation of a single power level charge and discharge curve, chosen as the nominal power, was conducted. Regarding the rest of the data validated with different power levels, it was possible to find a regression fit for each, based on the variation of the three extracted points from the curves but keeping all the other variables constant. Below, the approach is detailed.

***4.2.1. Battery behaviour representation for a single power level.*** The voltage curve was profiled and used for later model validation for the nominal inverter power level, 3300 W, over the defined SOC range and at a constant ambient temperature of 20°C (measured). For both experimental curves of charge and discharge – the contrary to the manufacturer curves as the modified Shepherd’s model defined – the three extracted points of voltage and energy capacity are hereinafter enunciated for the charge in Equation (7),


$$\;\{\begin{array}{c} \left(V_{full},Q_{full})=(60.90,169\right)\\ \left(V_{exp},Q_{exp})=(57.35,Q_{full}\times n_{\exp\_c}\right)\\ \left(V_{nom},Q_{nom})=(52.60,Q_{full}\times n_{nom}\right) \end{array}\;(7)$$

And discharge in Equation (8),


$$\;\{\begin{array}{c} \left(V_{full},Q_{full})=(60.90,169\right)\\ \left(V_{exp},Q_{exp})=(58.50,Q_{full}\times n_{\exp\_d}\right)\\ \left(V_{nom},Q_{nom})=(54.60,Q_{full}\times n_{nom}\right) \end{array}\;(8)$$

Where *n*_exp_ *c*_ is a percentage of the *Q _full_* to obtain the value of *Q _exp_* in the case of charge, and *n*_exp_ *d*_ in the case of discharge. The *n _nom_* is a percentage of the *Q _full_* to obtain the value of *Q _nom_*. In the case of the discharge state, the same percentage is given both for charge and discharge.

The values of Eq. (7) and Eq. (8) were obtained by implementing an iterative cycle. The three values of voltage should obey three conditions: they should be different from each other, obey the order *V _full_* > *V _exp_* > *V _nom_*, and should be in the range of 49 to 61 V. The iterative cycle is initiated with three guessed values made variable within the range of centesimal digits. The iterative cycle tests different values among the conditions referred, and the obtained curves are then compared with the experimental curve, where the one with the minor error compared with experimental values is used, and the guessed three points (V, Q) are its the correspondent values (presented in Eq. (7) and (8) for inverter nominal power). Then, the correspondent energy capacity ( *Q _full_*, *Q _exp_*, *Q _nom_*) for that voltage value is obtained. The same procedure took place for the attributed values *n*_exp_ *c*_, *n*_exp_ *d*_ and *n*_*nom*_ but now within the 0.1 to 1 range.

**Figure 11.**
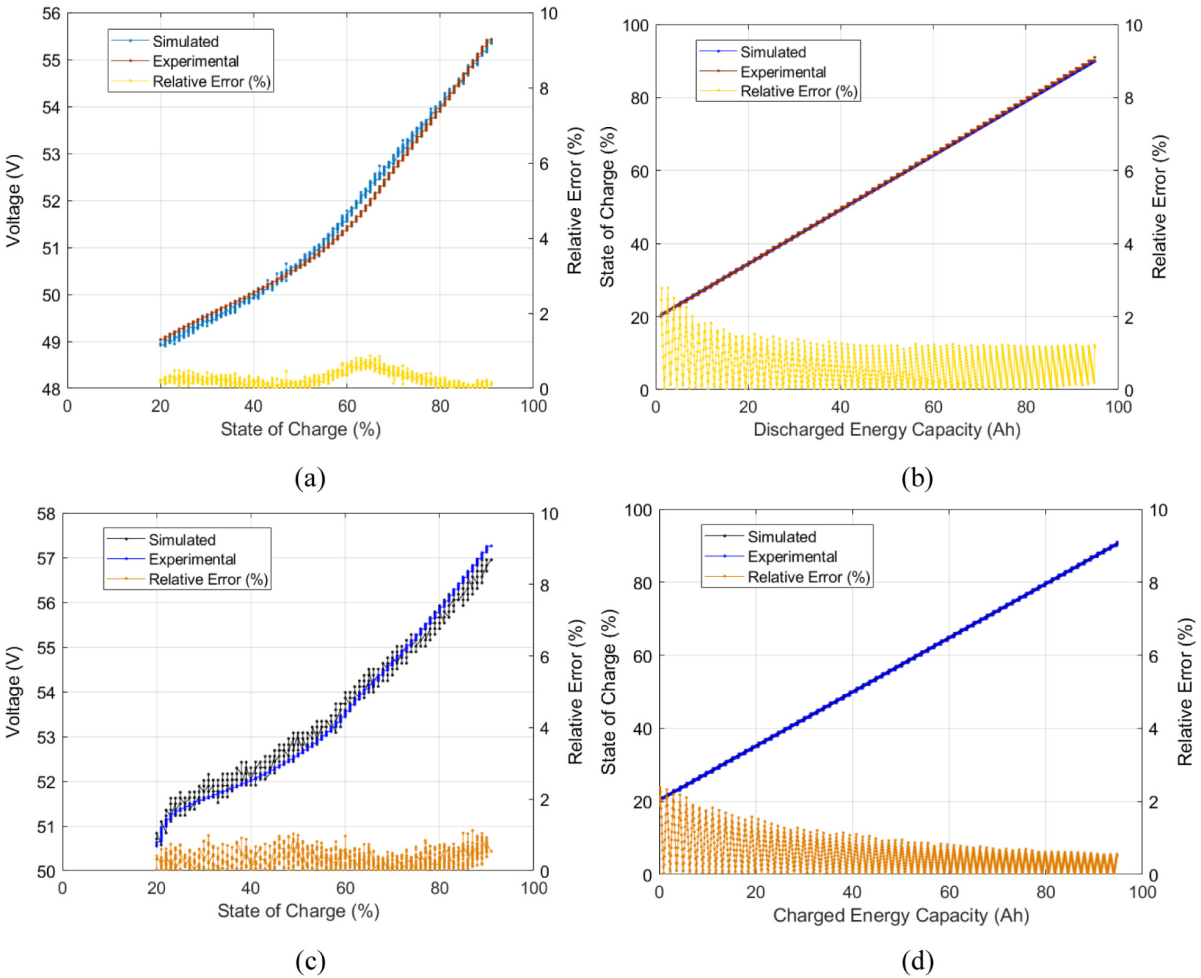
Model validation simulated vs experimental results for the 3300 W level of power: ( **a**) discharge voltage- State of Charge (SOC) curve, ( **b**) discharge SOC-energy capacity curve, ( **c**) charge voltage-SOC curve, and ( **d**) charge SOC-energy capacity curve.

This power level’s charge and discharge voltage curve presented a root mean square error (RMSE) of near 0.1 V and a maximum relative error of near 1.0 %. The obtained SOC values presented a maximum relative error of near 2.5 %. The experimental and simulated voltage curves for this power level are shown in Figure 11.

***4.2.2. Battery behaviour representation for all power levels (regression fit).*** Figure 11 shows the battery voltage curve over the different states of charge and SOC vs energy capacity of a single power level. For an overall battery description, distinct experimental power level curves should be tested within the simulated model. For the model to describe the different operating power levels of the battery, the three points of voltage and energy capacity (see Equation (7), Equation (8) and Figure 2) correspond to the experimental charge and discharge curves of each power level (or different current level), should be extracted (guessed). In that sense, the following approach aims to adjust the simulated model curves of each power level to the experimental results obtained, as Equation (9) indicates,


$$\;\{\begin{array}{c} {\left(V_{full},Q_{full}\right)}_{power\_level(soc)}=(V_{full}+Y,Q_{full}+Z)\\ {\left(V_{exp},Q_{exp}\right)}_{power\_level(soc)}=(V_{exp}+Y,Q_{exp}+Z)\\ {\left(V_{nom},Q_{nom}\right)}_{{power}_{level(soc)}}=(V_{nom}+Y,Q_{nom}+Z) \end{array}\;(9)$$

**Figure 12.**
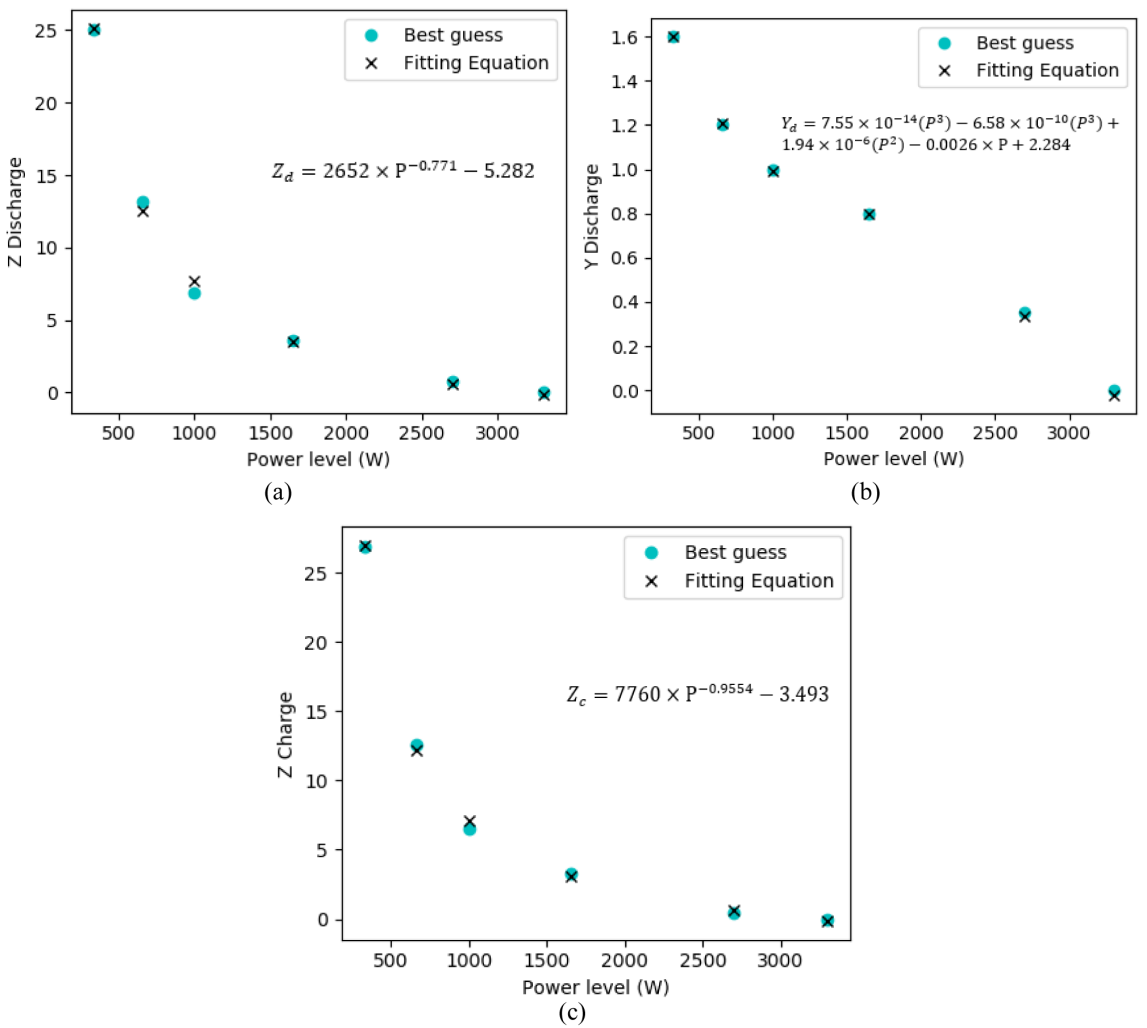
Y and Z regression fitting values obtained for the charge and discharge operating states, in function of the power levels of the battery operation.

Equation (9) was used to check the existence of a relationship of Y and Z with the extracted points (guessed) that better describe each power level experimental data. As firstly made to the first power level ( Section 4.2.1), points from the experimental curves of each power level were guessed, and afterwards, the values of Y and Z that better fit that data are obtained. A curve-fitting on the best-guessed values that described the battery’s experimental data was achieved with the help of the MATLAB curve fitting tools (also existing in alternative software, such as the open-sourced Python), both for charge and discharge operation state. Through the curve fitting, it was possible to find a function that describes the values of Y and Z that should be added/subtracted to the initial guessed three points of the curves. The functions’ representations are shown in Figure 12.

The guessed values that better described the three extracted points were compared with the best-fit function in these figures. The determination of Y and Z for both charge and discharge states offer, in this way, a full battery behaviour description within the experimental values obtained. To observe the voltage regression fit accuracy of the simulated values against the experimental acquired values, Table 5 and Table 6 present the RMSE and maximum relative errors of each fitting point with its correspondent experimental value for discharge and charge operating states, respectively. The obtained regressions allow the battery’s description through its distinct operation states in the regular operating conditions. The state of charge at each of the power level obtained values can be observed in Table 7. The developed model is made available in *Software availability* ^46^.

**Table 5.** Comparison of the simulated against experimental voltage values on discharge state, and respective errors (root mean square error (RMSE) and minimum and maximum relative errors). Where, *n*_exp_ *d*_ is a percentage of the *Q*_*full*_ to obtain the value of *Q*_*exp*_ in the case of discharge curve; *n*_nom_ is a percentage of the *Q*_*full*_ to obtain the value of *Q*_*nom*;_ *Y*_*d*_ and *Z*_*d*_ are the values to be added to the voltage and energy capacity, respectively, extracted discharged curves.

Power level (W)	*n*_exp_ *d*_	*n*_*nom*_	*Y*_*d*_	*Z*_*d*_	RMSE error (V)	Maximum Relative Error (%)
330	0.77	0.98	1.60	25.1	0.74	4.81
660			1.21	12.5	5.51	5.68
1000			0.99	7.65	1.28	3.11
1650			0.80	3.51	0.48	2.12
2700			0.35	0.73	0.05	1.17
3300			0.01	0.13	3.84	4.78

**Table 6.** Comparison of the simulated against experimental voltage values on charge state, and respective errors (root mean square error (RMSE) and minimum and maximum relative errors). Where, *n*_exp_ *c*_ is a percentage of the *Q*_*full*_ to obtain the value of *Q*_*exp*_ in the case of charge curve; *n*_nom_ is a percentage of the *Q*_*full*_ to obtain the value of *Q*_*nom*_; *Y*_*c*_ and *Z*_*c*_ are the values to be added to the voltage and energy capacity, respectively, extracted charged curves.

Power level (W)	*n*_exp_ *c*_	*n*_*nom*_	*Y*_*c*_	*Z*_*c*_	RMSE error (V)	Maximum Relative Error (%)
330	0.60	0.98	0.00	26.9	1.34	5.57
660			0.00	12.2	5.27	6.74
1000			0.00	7.07	1.06	2.75
1650			0.00	3.05	1.02	3.02
2700			0.00	0.60	0.47	3.78
3300			0.00	0.12	0.10	1.61

**Table 7.** Comparison of the simulated against the experimental State of Charge (SOC) values errors (root mean square error (RMSE) and minimum and maximum relative errors).

Operating state	Charge		Discharge	
Power level (W)	RMSE error (V)	Maximum Relative Error (%)	RMSE error (%)	Maximum Relative Error (%)
330	0.78	3.12	0.60	3.90
660	0.37	2.97	0.25	3.67
1000	0.30	6.53	0.31	3.60
1650	1.15	2.61	0.48	5.02
2700	0.20	2.62	0.79	6.02
3300	1.05	2.44	0.30	6.82

## 5. Discussion

In this work, a commercial LIB battery pack was the object of experimental tests through its complete charge and discharge at constant levels of a power testing plan. The manufacturer data helped to track the operating range limits and, in the testing, the definition of the overall condition. The establishment of inverter communication allowed the battery testing under controlled conditions. For that reason, the testing output is influenced by this unit. The LIB testing results, which enabled the energetic performance calculation presented in Table 4 and Figure 9, follow the expected results for the tested technology ^49, 50^. The values obtained for the inverter efficiencies are close to the manufacturer-provided data, with a calculated STD of 2.7 %.

A LIB model is commonly found in literature, mostly based on the MATLAB/Simulink pre-existing model ^27, 29, 30, 51^. The reproduction/justification of the model to be applied for a stationary application which could fit the obtained experimental data was not found. Thus, in this work, the authors based the modelling approach on one of the modified Shepherd’s models and adapted it to describe the real-time battery dynamic behaviour of the battery integrated into the microgrid.

The results obtained within the model approach used show satisfactory adequacies for all the values experimentally obtained with the LIB. In the discharge state, the higher voltage RMSE is 5.51 V, and the maximum relative error (MRE) is 5.68 %. The MRE obtained for the SOC is about 6.82 %. In the charge state, the highest RMSE for voltage is 5.27 V and an MRE of 6.74 %. The MRE obtained for SOC is 6.53 %. Given the equation fitting and the experimental data errors obtained, generally, the model describes the battery behaviour. The standby operation (self-discharge and inverter auxiliary consumption) will also be considered in future work for the final complete model.

The temperature effects were not fully considered in this approach. The higher the charge and discharge rates and ambient temperatures, the higher the internal resistance and generally, more heat is generated by the Joule effect. In this case, voltage and energy capacity are affected. The lack of details regarding this present battery composition makes it more challenging to include parameters that could describe those effects. The battery is operated under a controlled ambient temperature range, and the authors believe that the obtained figures for the curves include the slight temperature variations within the obtained errors. Moreover, the temperature dependence variation within the series resistance impacts LIB lifetime. This phenomenon will be approached in further research by considering the model describing the battery behaviour over time (to be used in future energy and economic assessments) and including temperature and SOC effects in the resistance calculation and the temperature and cycling effects in the calculation of battery energy capacity.

The achieved model describes, with small computational effort and fast runtime, the main characteristics of the battery, which facilitates its application within larger models of energy management strategies. The model is validated with different power levels and experimental battery data, improving its modelling of the battery behaviour with variable input profiles.

Similarly to most commercially available LIBs, the LIB manufacturer does not provide information regarding the internal construction, missing information such as the number of cells, parallel and series connections, voltage equalization algorithms, or temperature control algorithms. Missing important information limits the modelling accuracy, generating a higher error in the simulation results. Nevertheless, the maximum error obtained is considered low, being the model accurate to represent the battery performance, even with relevant battery data not being provided by the manufacturer.

## 6. Conclusions

This work applies and validates a model to a 9.80 kWh (189 Ah) lithium-ion commercial battery pack behaviour – voltage-current curves, energy capacity and SOC profiles with real-time variation – to give a potential modelling application to optimization and predictive microgrid programming control (including additional assets and corresponding models, such as PV systems), specifically for commercial and residential applications.

The correspondence between the general voltage-current models and the operating conditions matching the battery is usually a complex task. In this work, a LIB and inverter experimental setup was built to characterize performance and behaviour with precision monitoring. Communication was established with the battery inverter, enabling it to send real-time commands and get readings. This setup allowed performing the necessary characterization tests under real operating conditions.

Several curve fittings representing the battery behaviour under different operating states with a lower error were obtained. Charge and discharge errors were calculated and observed in Table 5, Table 6, and Table 7, with a maximum relative error of 6.74 % for simulated voltage.

Future work development will include applying this model within a larger simulation model, considering other systems present in the residential and commercial sectors. Further development should also include an ageing model and an energy management strategy, contributing to the technical and economic evaluation. Lithium-ion battery technology will continue to increase within its typical application in the automotive market, with the expectation that the technology will also be used in a second market for stationary applications. Accurate models for stationary application of LIB will provide significant advantages to the market uptake, in particular, for the residential and commercial sectors.

## Data availability

### Underlying data

Zenodo: Lithium-ion battery charge and discharge testing data - current, voltage, soc, ta - at constant levels of power. https://doi.org/10.5281/zenodo.5196334 ^45^.

This project contains the following underlying data:

– Lithium_Ion_Battery_Testing_Data.csv (this dataset was used in the composition of the lithium-ion battery testing and modelling validation. The file contains the charge and discharge testing acquisition data - current, voltage, soc, ta - at constant levels of power. The legend of the text is given in the final column “BI” of the .csv file.)

Data are available under the terms of the Creative Commons Attribution 4.0 International license (CC-BY 4.0).

## Software availability

Source code available from: https://github.com/catSelof/Batteries

Archived source code at time of publication: https://doi.org/10.5281/zenodo.5814884 ^46^

License: LGPL-2.1

## Ethics and consent

Ethical approval and consent were not required.
