# Supplementary material for: Validation of a lithium-ion commercial battery pack model using experimental data for stationary energy management application
Source: Open Res Eur. 2022 Jun 10;2:15. Originally published 2022 Feb 3. [Version 2] doi: 10.12688/openreseurope.14301.2 (PMC10445895; doi:10.12688/openreseurope.14301.2)
Supplement: Peer review history

# Peer review: Reviewer response for version 2

**DOI:** 10.21956/openreseurope.16071.r29510

**Published:** 2022-07-04

**Authors:** Yinghao Shan

**Affiliations:** 1 College of Information Science and Technology, Donghua University, Shanghai, China

My previous comments have been fully responded.

Is the study design appropriate and does the work have academic merit?

Partly

Is the work clearly and accurately presented and does it cite the current literature?

Yes

If applicable, is the statistical analysis and its interpretation appropriate?

Partly

Are all the source data underlying the results available to ensure full reproducibility?

Partly

Are the conclusions drawn adequately supported by the results?

Partly

Are sufficient details of methods and analysis provided to allow replication by others?

No

Reviewer Expertise:

BESS modeling and control

I confirm that I have read this submission and believe that I have an appropriate level of expertise to confirm that it is of an acceptable scientific standard.

# Peer review: Reviewer response for version 1

**DOI:** 10.21956/openreseurope.15429.r29042

**Published:** 2022-04-29

**Authors:** Yinghao Shan

**Affiliations:** 1 College of Information Science and Technology, Donghua University, Shanghai, China

Title: Validation of a lithium-ion commercial battery pack model using experimental data for stationary energy management application

 Comments:

1. The main contributions are suggested to be clearly listed in the Introduction. Besides, the motivation for modelling commercial battery packs should also be presented.
2. Will the modelling method be applicable to other commercial battery packs? Clarify the general academic contribution.
3. The axes in Fig. 7 are not labelled correctly with units.
4. The temperature effects were not fully considered in this approach. However, the temperature really influences the battery performance. Please explain.
5. The authors should provide more details about how to conduct the experiment to get the experimental data.

Is the study design appropriate and does the work have academic merit?

Partly

Is the work clearly and accurately presented and does it cite the current literature?

Yes

If applicable, is the statistical analysis and its interpretation appropriate?

Partly

Are all the source data underlying the results available to ensure full reproducibility?

Partly

Are the conclusions drawn adequately supported by the results?

Partly

Are sufficient details of methods and analysis provided to allow replication by others?

No

Reviewer Expertise:

BESS modeling and control

I confirm that I have read this submission and believe that I have an appropriate level of expertise to confirm that it is of an acceptable scientific standard, however I have significant reservations, as outlined above.

# Response

**Published:** 2022-05-31

**Authors:** Ana Catarina Foles

**Affiliations:** University of Évora, Portugal

Dear Professor,

 As the corresponding author, I would like to take the opportunity to thank you for your helpful comments resulting from the revision of the work "Validation of a lithium-ion commercial battery pack model using experimental data for stationary energy management application". The suggestions offered were considered and performed to address the Professor's concerns better, hoping the revised manuscript would better suit this field of expertise. In the following, the authors enunciate the changes and refinements made in the revised work to the Professors' comments through a numbered list.

1. Thank you for the comment. We have added a paragraph to the Introduction (currently the 3rd paragraph) with the motivation details. We have explained this modelling type application's use and relevance in this field of research.
2. The authors believe the approach can be reproducible to other battery packs to model the lithium-ion battery performance compared to experimental results. The academic contribution is further detailed in Section 2.2. of this work (in the penultimate paragraph).
3. Figure 7 from the previous version is currently Figure 8. The axes were corrected.
4. In Section 5 – Discussion – the fourth paragraph was extended to better approach the theme. In the case of the present work, the authors believe it fulfils the proposed modelling objectives based on the existing LIB modelling approaches (referenced in the bibliography).
5. Figure 5 was added to detail the realization of the experiment and data registering to better address that phase of the work. The experiment is focused on four main steps: the existence of physical equipment: battery compatible with an inverter that allows the modbus communication; the communication establishment with the inverter through modbus TCP/IP; the execution of complete charge and discharge cycles (considering the chosen depth of discharge), controlling different points of the AC power setpoint; and the execution of real-time measurements of current, voltage, SOC, temperature, and their data acquisition and file saving.

The authors hope the revised manuscript better suits the field of research and want to sincerely thank you for your interest in our research, allowing us to revise our work.

 Sincerely,

 Ana Catarina Foles

# Peer review: Reviewer response for version 1

**DOI:** 10.21956/openreseurope.15429.r28534

**Published:** 2022-02-23

**Authors:** Eduardo López

**Affiliations:** 1 Instituto Nacional de Técnica Aeroespacial - INTA (National Institute of Aerospace Technology), Mazagón, Spain

The article proposes the application of a model for lithium ion batteries in stationary applications, and its experimental validation from data obtained in tests at different power levels.

 The commercial battery system is integrated with an inverter and solar photovoltaic (PV) system as part of a microgrid. The battery and the inverter are fully characterized through the implementation of a testing protocol aiming at better describing the battery performance. Then, a battery model is built upon both the existent LIB description and experimental fitting regression, for real-time predictive optimization control development. Considering the predetermined efficiency of the inverter, the model allows to obtain the voltage curve, the series resistance (i.e., to describe instantaneous voltage drop/rise and transients), and the state of charge (SOC) and/or energy capacity, based on the current input. The developed model is validated through the comparison with the experimental results.

 The applied methodology and the experimental procedure is clear and convincing. The results show that the maximum error obtained is considered low, indicating the model accuracy for describing the battery performance, even with important battery data

 not being provided by the manufacturer.

 However, there are some minor comments that can help to better understand the application of the model to this particular case. For example, it is not clear how the values of the Equations 7 and 8 are obtained from the experimental curve corresponding to the 3.3 kW power level test. It should be advisable to include such charge-discharge curve and mark on it the values of V _full_, Qf _ull_, V _exp_, Q _exp_, V _nom_ and Q _nom_, as well as the assumptions made to estimate the percentages ne _xp_c_ and n _exp_d_ provided in Tables 5 and 6.

 Moreover, it should be advisable to explain more deeply how the SOC has been calculated to guarantee that the tests start at 20%, taking into account that if the Equation 5 is used, it would be necessary to know the capacity of the battery below this lower limit.

Is the study design appropriate and does the work have academic merit?

Yes

Is the work clearly and accurately presented and does it cite the current literature?

Yes

If applicable, is the statistical analysis and its interpretation appropriate?

Yes

Are all the source data underlying the results available to ensure full reproducibility?

Yes

Are the conclusions drawn adequately supported by the results?

Yes

Are sufficient details of methods and analysis provided to allow replication by others?

Partly

Reviewer Expertise:

Energy storage, microgrids

I confirm that I have read this submission and believe that I have an appropriate level of expertise to confirm that it is of an acceptable scientific standard.

# Response

**Published:** 2022-05-31

**Authors:** Ana Catarina Foles

**Affiliations:** University of Évora, Portugal

Dear Professor,

 As the corresponding author, I would like to take the opportunity to thank you for your helpful comments resulting from the revision of the work "Validation of a Lithium-ion commercial battery pack model using experimental data for stationary energy management application". The suggestions offered were considered and performed to address the Professor's concerns better. In the following, the authors enunciate the corresponding changes and refinements made in the revised paper.

 To what concerns the values obtained in Equations 7 and 8, the third paragraph of Section 4.2.1 was detailed to explain better the approach used to achieve the presented values, namely energy capacities, voltages, and percentages (V, Q and n). Figure 2 represents the overall model values visually. The charge-discharge curve extracted points are already presented in the equations.

 To what concerns the SOC calculation and Equation 5, the Q represents the total energy capacity of the battery, in Wh. In that sense, the first iteration (t) corresponds to a value of 20 %. In the second iteration, 20% is the previous iteration state of charge (SOC(t-1)), and Q is the total energy capacity of the battery (constant). We believe we had a poor explanation, and in that sense, the authors added the word "total" to the sentence "Where Q is the energy capacity of the battery", below Equation 6.

 The authors hope the revised manuscript will better suit the field of research and want to sincerely thank you for your interest in our research, allowing us to revise our work. Thank you very much for this revision. 

 Sincerely,

 Ana Catarina Foles
